# Hsa_circ_0020850 promotes the malignant behaviors of lung adenocarcinoma by regulating miR-326/BECN1 axis

**DOI:** 10.1186/s12957-021-02480-3

**Published:** 2022-01-10

**Authors:** Xiaoju Li, Shengtian Su, Dan Ye, Zhigao Yu, Wenjing Lu, Liang Liu

**Affiliations:** Department of Oncology, Xiantao First People’s Hospital of Yangtze University, No. 29, Middle Section of Mianzhou Avenue, Xiantao, 433000 Hubei Province People’s Republic of China

**Keywords:** circ_0020850, miR-326, BECN1, Lung adenocarcinoma

## Abstract

**Background:**

Circular RNAs (circRNAs) are a novel type of endogenous RNAs and play vital roles in lung adenocarcinoma. However, the function and underlying mechanism of circ_0020850 in lung adenocarcinoma remain unknown.

**Methods:**

The levels of circ_0020850, microRNA-326 (miR-326), and Beclin1 (BECN1) were analyzed by real-time quantitative polymerase chain reaction and western blot analyses. The migration and invasion were determined by wound healing and transwell assays, respectively. Colony formation assay was used to assess cell proliferation ability. The angiogenic ability was analyzed by Matrigel angiogenesis assay. The apoptosis rate was calculated by flow cytometry assay. Dual-luciferase reporter, RNA immunoprecipitation (RIP), and RNA pull-down assays were conducted to confirm the interaction relationship among circ_0020850, miR-326, and BECN1. A xenograft mice model was established to assess the role of circ_0020850 in vivo.

**Results:**

We found that circ_0020850 was obviously overexpressed in lung adenocarcinoma tissues and cells. Knockdown of circ_0020850 inhibited migration, invasion, proliferation, and angiogenesis but induced apoptosis in lung adenocarcinoma cells in vitro, as well as curbed tumor growth in vivo. MiR-326 was a target of circ_0020850, and knockdown of miR-326 abolished the suppression effect of circ_0020850 on the malignant behaviors of lung adenocarcinoma cells. Additionally, miR-326 could negatively regulate BECN1 expression, thereby regulating lung adenocarcinoma cell phenotypes. Importantly, circ_0020850 could directly bind to miR-326 and thus relieve miR-326-mediated inhibition on BECN1.

**Conclusion:**

Circ_0020850 promoted the malignant development of lung adenocarcinoma by regulating miR-326/BECN1 axis, indicating that circ_0020850 might serve as a promising target for the diagnosis and treatment of lung adenocarcinoma patients.

**Supplementary Information:**

The online version contains supplementary material available at 10.1186/s12957-021-02480-3.

## Background

Lung cancer is one of the foremost reasons for tumor-associated mortality in this world [1]. Adenocarcinoma is the most common histological subtype of primary lung cancer [2]. In China, both the incidence and mortality rates of lung cancer are the highest and remain growing rapidly in recent years [3, 4]. Despite great improvements in lung adenocarcinoma diagnosis and treatment, the prognosis of lung adenocarcinoma patients remains poor due to the lack of early diagnosis target and its high recurrence and metastasis rate [5, 6]. Hence, it is urgently needed to seek novel molecular therapeutic or prognostic targets for lung adenocarcinoma.

Circular RNAs (circRNAs) are one group of new RNA molecules with a closed continuous loop but lacking 5′ (cap) and 3′ (polyadenylation) ends [7]. Several researches have reported the dysregulation of circRNA in multiple cancers, including lung cancer [8]. Besides, mounting studies have identified a circRNA-miRNA-mRNA regulatory network in cancers [9]. Current research indicated that circRNA could function as a critical regulator in cancers through sponging microRNA (miRNA) to inhibit miRNA activity and subsequently upregulate target mRNA expression [10, 11]. Hsa_circ_0020850 is coded by the nucleoporin 98 and 96 precursor (NUP98) gene and located on chr11:3,697,738-3,789,974. Interestingly, circ_0020850 was reported to act as a cancerogenic circRNA in hematological cancers [12] and renal cell carcinoma [13]. However, the detailed functions of circ_0020850 in lung cancer are lacking. Here, we aimed to investigate the function and regulatory mechanism of circ_0020850 in lung adenocarcinoma.

MiRNAs, a class of endogenous and non-protein-coding small RNAs [14], are vital to the regulation of posttranscriptional level of target mRNAs by interaction with 3′ untranslated regions (3′UTRs) [15]. Previous reports showed that miR-326 played key roles in the pathogenesis of various human cancers [16, 17]. Increasing miRNA genomic profiles reported the prognostic value of multiple miRNAs for patients with lung adenocarcinoma [18, 19], including miR-326 [20]. The public bioinformatics database presents that miR-326 is a potential target of circ_0020850, suggesting that miR-326 may be regulated by circ_0020850. Interestingly, miR-326 is predicted to have binding sites with Beclin1 (BECN1) 3′UTR. BECN1 has been confirmed to modulate apoptosis and autophagy in cancer cells [21, 22]. Additionally, BECN1 knockdown largely improved the therapeutic effects of chemistry medicines in cancer treatment [23, 24]. It is necessary to be addressed whether miR-326-mediated BECN1 is involved in circ_0020850 regulatory networks.

In this study, we detected the expression level of circ_0020850 in lung adenocarcinoma tissues and cells, and confirmed that circ_0020850 was upregulated in lung adenocarcinoma tissues and cells. Based on the competing endogenous RNA (ceRNA) hypothesis, we also investigated the function of circ_0020850-mediated regulatory network in lung adenocarcinoma progression.

## Materials and methods

### Patient tissues

Thirty-seven pairs of lung tissues and adjacent normal tissues were collected from patients undergoing a surgical procedure at Xiantao First People’s Hospital of Yangtze University, and all the procedures got permission from the Ethics Committee of Xiantao First People’s Hospital of Yangtze University. The clinicopathological characteristics of patients are presented in Table 1, using chi square test here. Furthermore, written informed consents were offered by all patients. The removed tissues were promptly transferred to − 80 °C for subsequent study.

**Table 1:** Correlation between circ_0020850 expression and clinicopathological characteristics of lung adenocarcinoma patients

Clinical parameter	Low-circ_0020850 (*n*=19)	High-circ_0020850 (*n*=18)	*P*
Age (years)			
<60	7	10	0.254
>60	12	8	
Gender			
Male	11	9	0.630
Female	8	9	
Histological grade			
Low or undiffer	8	13	0.065
Middle or high	11	5	
TNM stages			
I and II	13	5	0.013^*^
III and IV	6	13	
Size			
≤3 cm	12	10	0.638
>3 cm	7	8	
Invasion depth			
T1 and T2	13	5	0.013^*^
T3 and T4	6	13	
Lymphatic metastasis			
Yes	7	13	0.031^*^
No	12	5	
Distant metastasis			
Yes	10	11	0.603
No	9	7	

*P<0.05

### Cell lines and cell culture

Human bronchial epithelioid cells (16HBE) and lung adenocarcinoma cells (A549 and HCC827) were provided by Nanjing Key Gen Biotech (Nanjing, China) and then cultivated in Dulbecco’s modified Eagle’s medium (Life Technologies, Scotland, UK) containing 10% (v/v) fetal bovine serum (FBS; Life Technologies) at 37 °C, 5% CO_2_.

### Quantitative real-time polymerase chain reaction (RT-qPCR)

Total RNA was prepared using Trizol reagent (Takara, Dalian, China) as recommended by the manufacturers. The extracted total RNA was reverse-transcribed into complementary DNA by reverse transcription kit (Takara) or microRNA Reverse Transcription Kit (Qiagen, Hilden, Germany). Expression levels of RNAs were detected by SYBR® qPCR Mix (Toyobo, Tokyo, Japan) under the Roche Light-Cycler (Roche, Basel, Switzerland) based on the 2^−ΔΔCt^ method. Glyceraldehyde-3-phosphate dehydrogenase (GAPDH) and RNA U6 were used as endogenous controls. In addition, part of total RNA was exposed to RNase R (3 U/mg; Geneseed, Guangzhou, China) for 30 min at 37 °C to assess the stability of circ_0020850 and NUP98. The primer sequences were showed: circ_0020850-forward 5′-TAAAGATCGCCTGGCTCAGT-3′ and circ_0020850-reverse 5′-GGTTGTAGCCTGGCCAAAT-3′; NUP98-forward 5′-CTCCACCACTAATTCAGGCTTT-3′ and NUP98-reverse 5′-GAGGCTGGTAGTCTGCTGATT-3′; miR-326-forward 5′-GCCGAGCCTCTGGGCCCTTC-3′ and miR-326-reverse 5′-CAGTGCAGGGTCCGAGGTAT-3′; BECN1-forward 5′-GGTGTCTCTCGCAGATTCATC-3′ and BECN1-reverse 5′-TCAGTCTTCGGCTGAGGTTCT-3′; U6-forward 5′-GTGCTCGCTTCGGCAGCACA-3′ and U6-reverse 5′-GGAACGCTTCACGAATTTG-3′; GAPDH-forward 5′-CATCCATGACAACTTTGGTA-3′ and GAPDH-reverse 5′-CGTTGGCAGTGGGGACACGG-3′.

### Transfection assay

Small interfering RNA (siRNA) targeting circ_0020850 (si-circ_0020850) or circ_0020850-overexpression vector (circ_0020850) were all designed by HanBio (Shanghai, China), with si-con and pCD5-ciR as negative controls. MiR-326 mimic (miR-326), miR-con, miR-326 inhibitor (in-miR-326), in-miR-con, BECN1-overexpression vector (BECN1), and empty vector (pcDNA) were synthesized by Sangon (Shanghai, China). In total, 100 nM of siRNA, 60 nM of miRNA mimic or inhibitor, or 1 μg of plasmid was transfected into A549 and HCC827 cells using Lipofectamine 3000 (Life Technologies) as per the manufacturing protocols.

### Cell migration and invasion assays

Wound healing assay was carried out to detect the migration of A549 and HCC827 cells. The transfected A549 and HCC827 cells (5 × 10^5^ cells/mL) were plated into the 6-well plates. After being cultured for 24 h, the attached cells were scratched with a sterile 200-μL micropipette tip to create a scratch wound, and the wound healing rate was reported under the inverted microscope (Mshot, Guangdong, China) after 0 and 24 h. All experiments were independently repeated three times.

Cell invasion was assessed by the transwell assay using the 24-well chambers with 8-μm pore size and pro-coated with Matrigel (BD Biosciences, Erembodegem, Belgium). In brief, transfected A549 and HCC827 cells (1 × 10^5^ cells/mL) in 200 μL serum-free medium were seeded into the upper chamber of transwell chambers, while the bottom chamber was supplemented with complete medium. After cultivation for 24 h, cells on the upper side of the membrane were removed, and the cells that remained adherent to the lower membrane surface were fixed in 4% paraformaldehyde and stained with 0.25% crystal violet (Beyotime, Shanghai, China) for 20 min, then photographed using a microscope (Mshot) at × 100 magnification. The cell number was counted in five random fields of view using the ImageJ software (NIH, Bethesda, MD, USA).

### Colony formation assay

For the colony formation assay, 500 cells were seeded into 6-well culture plates, gently shaken, and then incubated with complete medium containing 10% FBS for 2 weeks with three repetitions. After washing by phosphate-buffered saline, colonies were stained with 0.25% crystal violet (Beyotime). Colonies of more than 50 cells were scored under an inverted microscope (Mshot). And the efficiency of colony formation was calculated using the formula: colony formation rate (%) = (number of colonies/number of inoculating cells) × 100%.

### Tube formation assay

The tube formation analysis was conducted according to the previous research [25]. In total, 100 μL of human umbilical vein endothelial cells (HUVECs) was seeded into 96-well plates at 3 × 10^4^ cells per well coated with Matrigel (BD Biosciences) and then supplemented with 100 μL of conditional medium. The endothelial tubule formation was observed and photographed under the inverted microscope (Mshot), and the percentage of tube formation was calculated by ImageJ software (NIH).

### Flow cytometry

For analysis of cell apoptosis, A549 and HCC827 cells were collected after transfection for 48 h. Next, cell suspension (1 × 10^6^ cells/mL) of A549 and HCC827 cells was stained with Annexin V labeled with fluorescein isothiocyanate (FITC) and propidium iodide (PI) (BioVision, Milpitas, CA, USA) for 30 min in the darkness. The apoptotic cells were counted under the flow cytometer (BD Biosciences), and data were analyzed by AccuriC6 software (BD Biosciences).

### Dual-luciferase reporter assay

Three bioinformatics prediction databases, including CircBank (http://www.circbank.cn/), Starbase (http://starbase.sysu.edu.cn/), and Circular RNA interactome (http://circinteractome.nia.nih.gov/index.html), were used to predict target miRNAs of circ_0020850. The possible target genes of miR-326 were predicted by microT-CDS. To confirm the association between circ_0020850, miR-326, and BECN1. A549 and HCC827 cells were transfected with luciferase reporter vector harboring the wild type or mutant circ_0020850 fragment (circ_0020850-WT or circ_0020850-MUT) and type or mutant 3′UTR of BECN1 fragment (BECN1-3′UTR-WT or BECN1-3′UTR-MUT; all designed by Promega, Madison WI, USA) in the presence or absence of with miR-326. Luciferase activities were analyzed post-transfection for 48 h using the Dual-Luciferase Reporter Assay System (Promega, Madison WI, USA), and the activity of Renilla luciferase was used as an internal control.

### RNA immunoprecipitation (RIP)

RIP assay was carried out using a Magna RNA-binding protein immunoprecipitation kit (Sigma, Louis, MO, USA) according to the manufacturer’s instruction. A549 and HCC827 cells were washed with phosphate buffer solution (PBS) and then lysed in RIP buffer. Then cell lysates were incubated with magnetic beads coupled with Ago2 or IgG antibodies at 4 °C overnight, followed by the isolation of RNA for RT-qPCR assay.

### RNA pull-down assays

RNA pull-down assay was conducted using the Pierce Magnetic RNA–Protein Pull down Kit (Thermo Fisher Scientific) according to the manufacturer’s instructions. In brief, the biotin-labeled circ_0020850 probe was synthesized by RiboBio (Shanghai, China) and then incubated with cell lysates of A549 and HCC827 cells to pull down the candidate target miRNAs of circ_0020850. The RNA complex was then precipitated by conjugating with streptavidin magnetic beads. After elution, RNA level was assessed by RT-qPCR assay.

### Western blot assay

The tissues and cells were lysed in Radio-Immunoprecipitation buffer (Beyotime), and quantified by bicinchoninic acid (BCA) protein kit (Bio-Rad, Hercules, CA, USA). Then, an equal amount of proteins was separated by 10–12% sodium dodecyl sulfate polyacrylamide gel electrophoresis, and transferred onto the polyvinylidene difluoride membranes (Bio-Rad). After blocking with 5% non-fat milk, the membranes were incubated with anti-BECN1 (1:1000 dilution; Cell Signaling Technology, Cambridge, MA, USA) or β-actin (1:2000 dilution; Cell Signaling Technology) overnight at 4 °C. Appropriate horseradish peroxidase-conjugated secondary antibodies (1:2000 dilution; Cell Signaling Technology) were applied to detect labeled proteins. The protein bands were visualized with SuperSignal Ultra Chemiluminescent Substrate (Pierce, Rockford, IL, USA) on imaging system (Protein Simple, Santa Clara, CA, USA).

### Animal experiment

BALB/c nude mice (Beijing Vital River Laboratory, Beijing, China) were housed under specific pathogen-free conditions (temperature, 18–29 °C; relative humidity, 50–60%; free access to clean food and water; and lighting for 10 h). The nude mice were randomly separated into 3 groups (6 animals in each group). The sh-circ_0020850 group subcutaneously injected A549 cells was stably transfected with sh-circ_0020850 designed by HanBio (5 × 10^6^ cells/mice). Tumor growth was reported regularly and calculated using the formula: *V* = 1/2 × *ab*^2^ where “*a*” and “*b*” are the long and short diameter of the tumor, respectively. After 4 weeks, all mice were sacrificed by flowing CO_2_, and the animal experiment was performed in accordance with guidelines and approval from the Institutional Animal Care and Use Committee of Xiantao First People’s Hospital of Yangtze University. Immunohistochemistry (IHC) staining assay was used to assess Ki-67 level in xenograft tumor tissues as previously described [26]. In addition, anti-Ki-67 (1:800 dilution) was purchased from Cell Signaling Technology.

### Statistical analysis

Data were displayed as the mean ± standard deviation. Statistical analysis was carried out using Student’s *t* test (when two groups were compared) or analysis of variance (when > 2 groups were compared) followed by Bonferroni post hoc (SPSS 21.0; IBM, Somers, NY, USA). *P* value less than 0.05 was considered as a statistical difference. Pearson’s correlation analysis was used to determine the correlation among circ_0020850, miR-326, and BECN1. Overall survival rate was summarized by Kaplan-Meier analysis.

## Results

### Circ_0020850 was overexpressed in lung adenocarcinoma tissues and cells

**Fig. 1:**
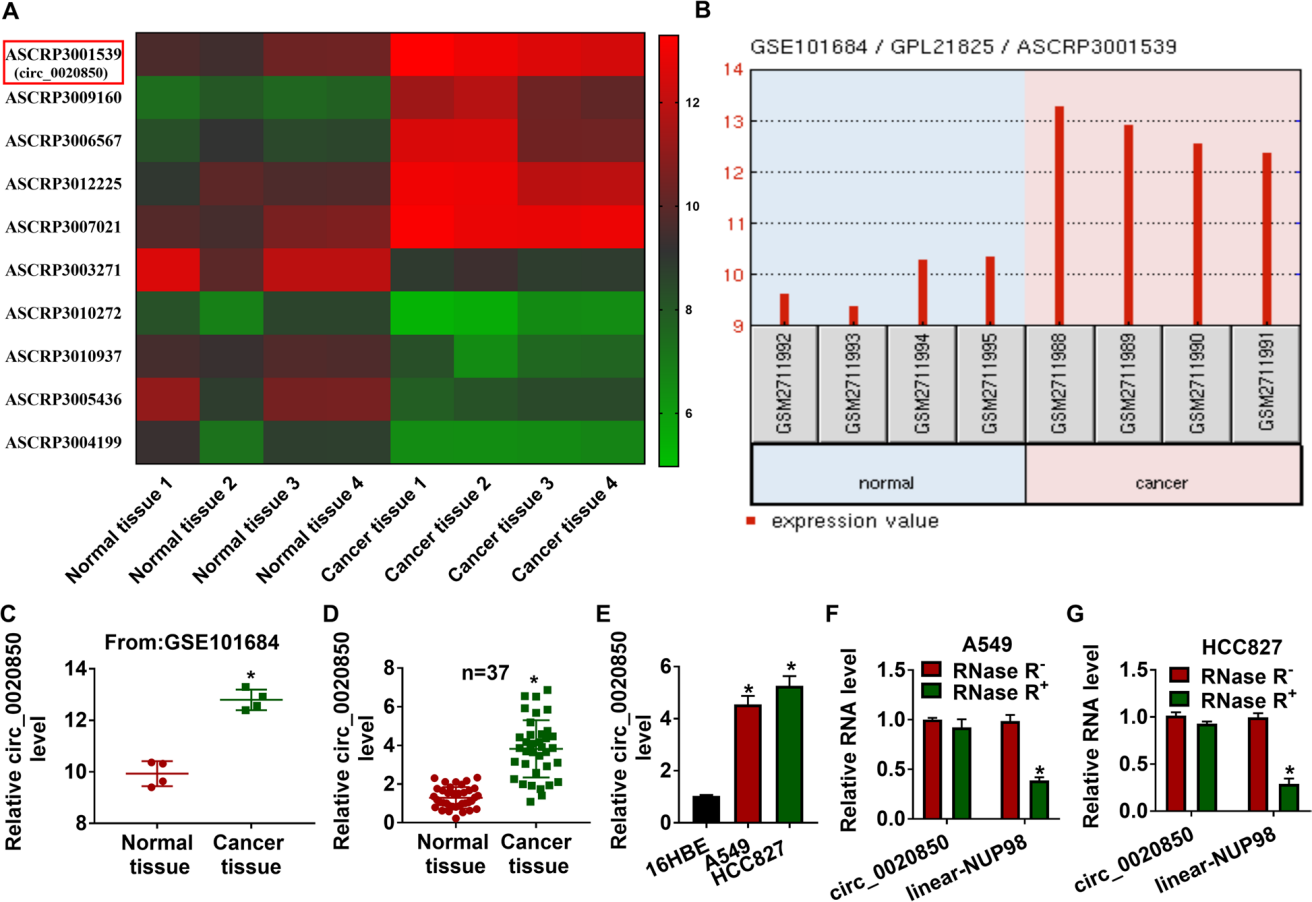
The expression level of circ_0020850 in lung adenocarcinoma tissues and cells. **A, B** The differentially expressed circRNAs were presented based on GSE101684 data. **C** GSE101684 data showed circ_0020850 level in lung adenocarcinoma tissues and normal tissues. **D, E** RT-qPCR was used to detect circ_0020850 level in lung adenocarcinoma tissues and cells, as well as in matched control groups. **F, G** The relative expression level of circ_0020850 and linear-NUP98 were assessed by RT-qPCR after treating with RNase R. *P < 0.05

The previous research suggested that circ_0020850 was overexpressed in lung adenocarcinoma tissues when compared with control tissues (Fig. 1A–C). Therefore, 37 lung adenocarcinoma tissues were collected for RT-qPCR in this study, and results of RT-qPCR suggested that lung adenocarcinoma tissues showed a higher expression of circ_0020850 than that in paired normal tissues (Fig. 1D). Also, circ_0020850 was also more upregulated in A549 and HCC827 cells than that in 16HBE cells (Fig. 1E). As circRNA is resistant to RNase R degradation, to verify the circular structure of circ_0020850, the RNAs extracted from A549 and HCC827 cells were treated with or without RNase R. As shown in Fig. 1F, G, RNase R digestion significantly decreased the level of linear-NUP98 rather than the circ_0020850, indicating that circ_0020850 could resist RNase R degradation (Fig. 1F, G). In addition, we further investigated the correlation between circ_0020850 expression and the clinical features of patients. As shown in Table 1, the expression level of circ_0020850 was closely associated with TNM stages (*P* = 0.032), tumor size (*P* = 0.003), and invasion depth (*P* = 0.032) of patients. Thus, circ_0020850 is a novel circRNA and is closely related to the prognosis of lung adenocarcinoma patients.

### Circ_0020850 knockdown inhibited migration, invasion, proliferation, and angiogenesis while it induced apoptosis of lung adenocarcinoma cells

**Fig. 2:**
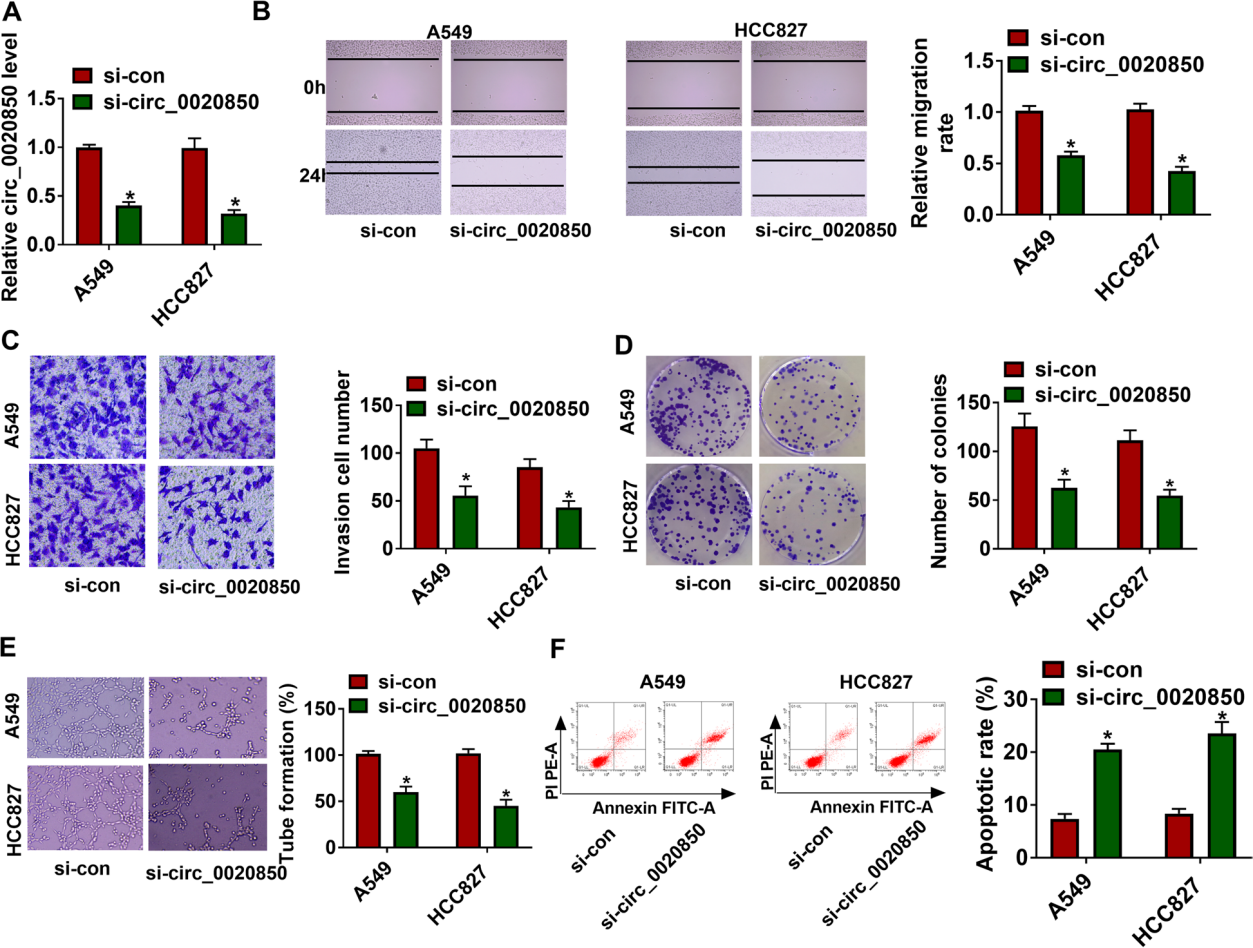
Influences of circ_0020850 silencing on migration, invasion, proliferation, angiogenesis, and apoptosis of lung adenocarcinoma cells. **A–F** A549 and HCC827 cells were transfected with si-con or si-circ_0020850. **A** The interference efficiency of si-circ_0020850 was analyzed by RT-qPCR. **B, C** The migration and invasion were measured by wound healing and transwell assays, respectively. **D** Colony-forming assay was performed to assess the effects circ_0020850 silencing on cell proliferation. **E** The matrigel angiogenesis assay was used to analyze the tube formation in A549 and HCC827 cells post-transfection. **F** The apoptosis rate was measured by flow cytometry assay. *P < 0.05

As circ_0020850 was upregulated in lung adenocarcinoma cells, we further investigated the function of circ_0020850 in lung adenocarcinoma cells by transfecting si-circ_0020850 into A549 and HCC827 cells. Circ_0020850 was obviously decreased in si-circ_0020850-transfected A549 and HCC827 cells in contrast with the cells transfected with si-con (Fig. 2A). Wound healing assay revealed that circ_0020850 knockdown impeded cell migration in A549 and HCC827 cells, while the invasion of A549 and HCC827 cells was suppressed by inhibition of circ_0020850 (Fig. 2B, C). The knockdown of circ_0020850 also decreased the colony numbers of A549 and HCC827 cells (Fig. 2D). Furthermore, tube formation assay uncovered that the angiogenesis ability of A549 and HCC827 cells was inhibited by circ_0020850 knockdown (Fig. 2E). Moreover, downregulation of circ_0020850 stimulated cell apoptosis in A549 and HCC827 cells (Fig. 2F). Thus, circ_0020850 regulated the multiple malignant behaviors of A549 and HCC827 cells, suggesting the key role of circ_0020850 in lung adenocarcinoma progression.

### Circ_0020850 targeted miR-326 in lung adenocarcinoma cells

**Fig. 3:**
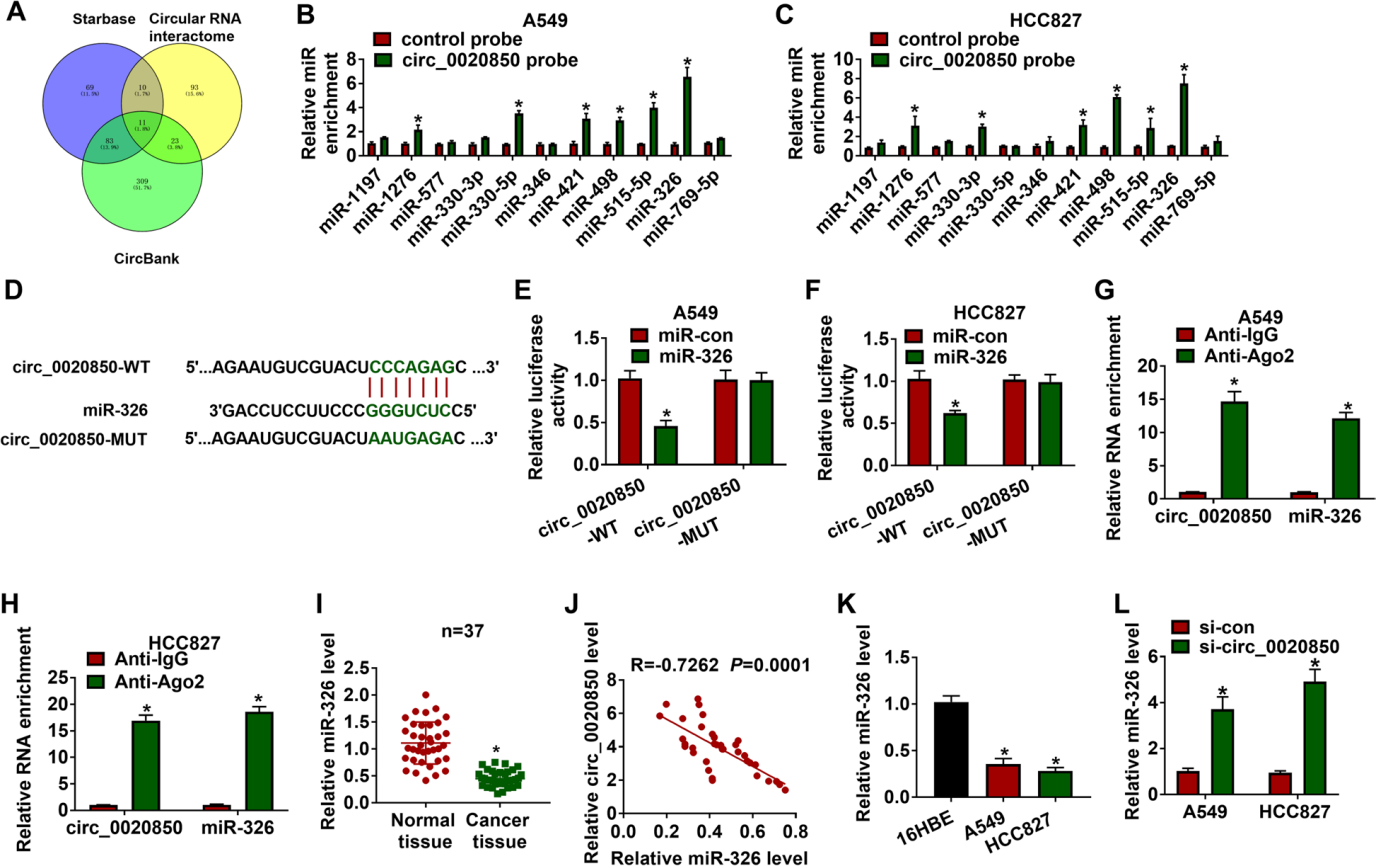
MiR-326 was a direct target of circ_0020850. **A** Schematic illustration showed the overlap of the target miRNAs of circ_0020850 based on bioinformatics analysis. **B, C** The relative levels of miRNA candidates were assessed by RT-qPCR in A549 and HCC827 cells after pull down by circ_0020850 probe. **D** The complementary sequences between miR-326 and circ_0020850 were presented. **E–H** The association between miR-326 and circ_0020850 was analyzed by dual-luciferase reporter and RIP assays. **I** The expression level of miR-326 was assessed by RT-qPCR assay in lung adenocarcinoma tissues and control. **J** The correlation between miR-326 and circ_0020850 was analyzed by Pearson’s correlation analysis. **K** RT-qPCR was used for examining miR-326 level in 16HBE, A549, and HCC827 cells. **L** The relative expression level of miR-326 was analyzed by RT-qPCR assay in A549 and HCC827 cells transfected with si-con or si-circ_0020850. *P < 0.05

Since circRNAs could serve as miRNA sponges to regulate the activation of miRNAs, we speculated that circ_0020850 may function as a miRNA sponge. Three bioinformatics databases were used for the screening of miRNAs that might target by circ_0020850. As shown in Fig. 3A, eleven target miRNAs (miR-1197, miR-1276, miR-577, miR-330-3p, miR-330-5p, miR-346, miR-421, miR-498, miR-515-5p, miR-326, and miR-769-5p) were predicted to be the target of circ_0020850. Among these miRNAs, RNA pull-down assay indicated that miR-326 was the most enriched miRNA (Fig. 3B, C). Thus, miR-326 was selected for further research. To verify the correlation between miR-326 and circ_0020850, circ_0020850 sequences which contained the wild type or mutant type miR-326 binding sites were constructed into the luciferase reporter vector (Fig. 3D). Besides, transfection of miR-326 significantly elevated the luciferase activity of circ_0020850-WT group in contrast with miR-NC transfection group, whereas it has little effect on circ_0020850-WT group (Fig. 3E, F). Besides, RIP assay uncovered that miR-326 and circ_0020850 were specifically enriched in the anti-Ago2 group rather than anti-IgG group, suggesting that miR-326 and circ_0020850 are recruited to the RNA-induced silencing complex (RISC) (Fig. 3G, H). Importantly, miR-326 was downregulated and was negatively correlated (*r* = − 0.4548, *P* = 0.0047) with circ_0020850 expression in 37 cases of lung adenocarcinoma tissues (Fig. 3I, J). Similarly, miR-326 expression showed a lower level in A549 and HCC827 cells compared with that in 16HBE cells (Fig. 3K). Furthermore, we found that circ_0020850 knockdown significantly increased the expression of miR-326 in A549 and HCC827 cells (Fig. 3L). Therefore, miR-326 was negatively regulated by circ_0020850 in lung adenocarcinoma cells.

### Circ_0020850 regulated migration, invasion, proliferation, angiogenesis, and apoptosis of lung adenocarcinoma cells by targeting miR-326

**Fig. 4:**
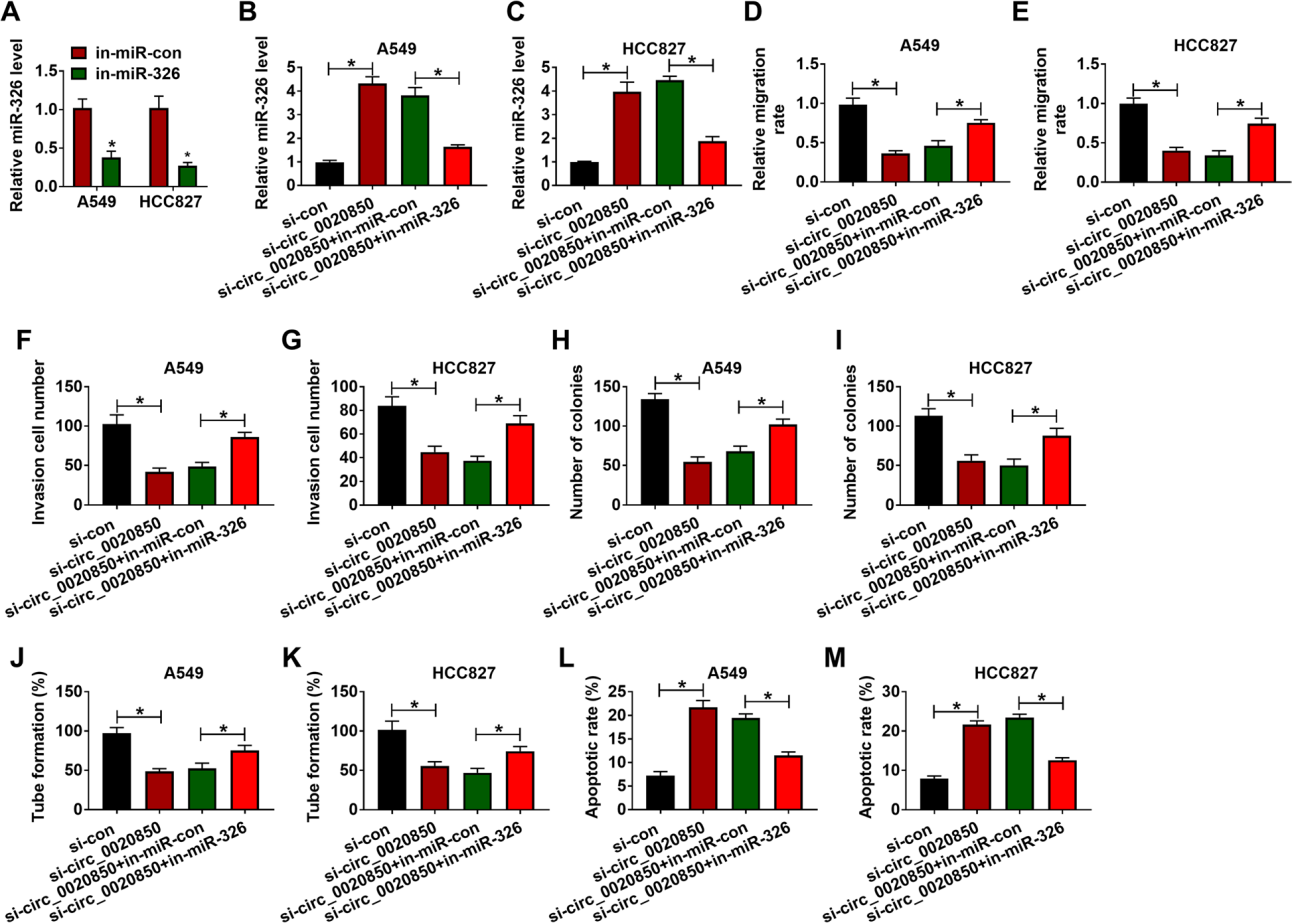
Knockdown of circ_0020850-induced effects on lung adenocarcinoma cells were abolished by inhibition of miR-326. **A** A549 and HCC827 cells were transfected with in-miR-326 to inhibit miR-326 expression. **B–M** A549 and HCC827 cells were transfected with si-con, si-circ_0020850, si-circ_0020850+in-miR-con, or si-circ_0020850+in-miR-326. **B, C** The expression level of miR-326 was analyzed by RT-qPCR. **D–G** The migration and invasion were assessed by wound healing and transwell assays, respectively. **H, I** The proliferation ability of A549 and HCC827 cells was analyzed by colony-forming assay. **J, K** The matrigel angiogenesis assay was conducted in A549 and HCC827 cells post-transfection. **L, M** The flow cytometry assay was carried out to assess apoptosis rate in A549 and HCC827 cells. *P < 0.05

To explore whether circ_0020850 regulated the biological behaviors of lung adenocarcinoma cells by targeting miR-326, loss-of-function experiments were conducted in A549 and HCC827 cells. MiR-326 was decreased by half in A549 and HCC827 cells with in-miR-326 transfection (Fig. 4A). And circ_0020850 knockdown elevated the level of miR-326 by four times, while co-transfection of in-miR-326 abolished the upregulation effect of si-circ_0020850 on miR-326 expression in A549 and HCC827 cells (Fig. 4B, C). Next, we further investigated the function of miR-326 in circ_0020850-mediated lung adenocarcinoma progression. The inhibitory effects of si-circ_0020850 on migration (Fig. 4D, E) and invasion (Fig. 4F, G, Supplementary Fig. 1 A) of A549 and HCC827 cells were partly rescued by miR-326 inhibitor. Besides, miR-326 inhibitor also overturned the suppression effect of si-circ_0020850 on colony formation and tube formation in A549 and HCC827 cells (Fig. 4H–K). Furthermore, the knockdown of miR-326 also protected A549 and HCC827 cells from circ_0020850 inhibition-induced apoptosis (Fig. 4L, M, Supplementary Figure 1B). Taken together, circ_0020850 regulated migration, invasion, proliferation, angiogenesis, and apoptosis by sponging miR-326 in lung adenocarcinoma cells.

### MiR-326 regulated BECN1 expression in lung adenocarcinoma cells

**Fig. 5:**
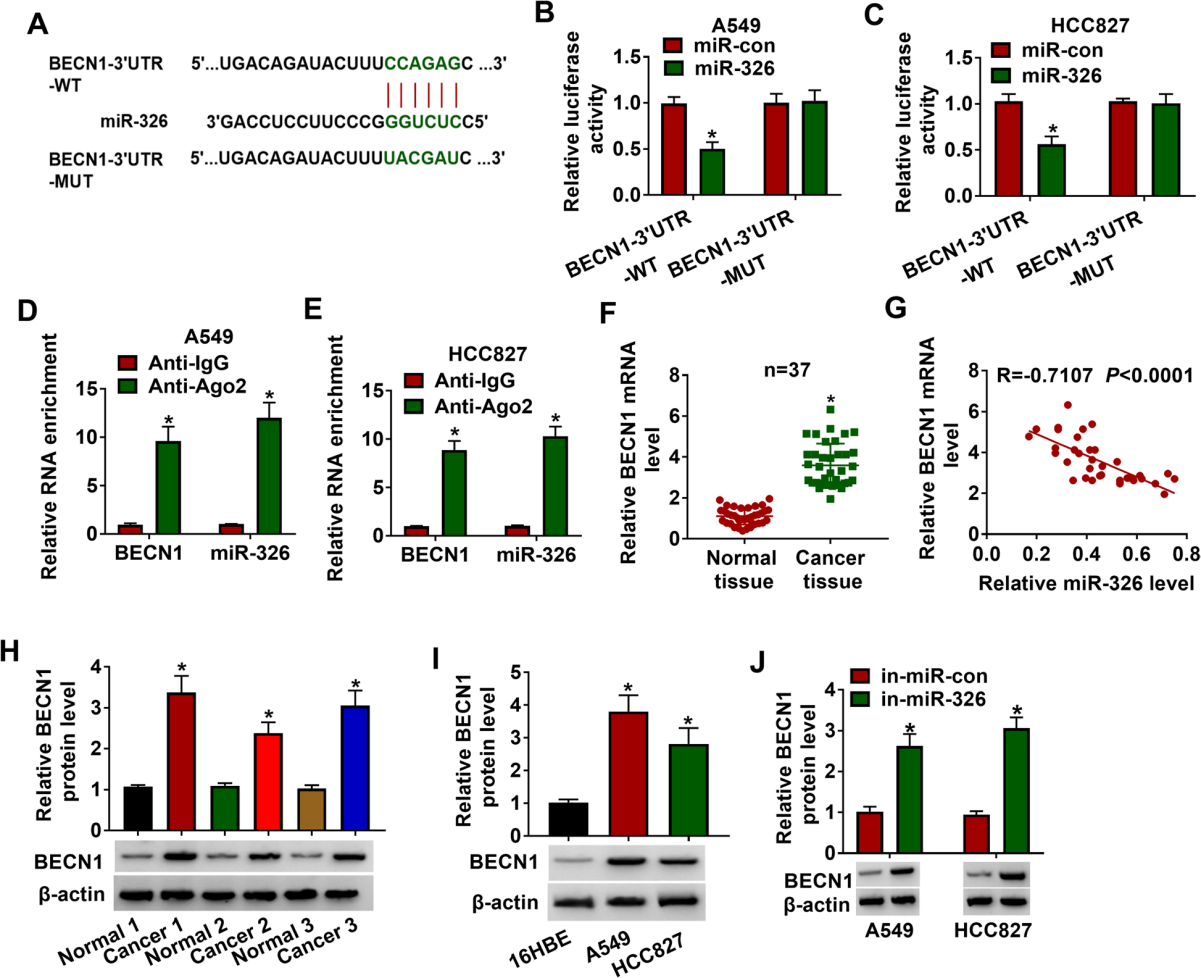
BECN1 was a functional gene of miR-326. **A** The binding regions between BECN1 and miR-326 were displayed. **B–E** Dual-luciferase reporter and RIP assays were conducted to analyze the association between BECN1 and miR-326. **F** The mRNA expression of BECN1 was determined by RT-qPCR in lung adenocarcinoma tissues and control. **G** The correlation between BECN1 and miR-326 was conducted by Pearson’s correlation analysis. **H, I** Western blot assay was used to examine BECN1 level in lung adenocarcinoma tissues and cells along with matched controls. **J** After transfection with in-miR-con or in-miR-326, the protein expression of BECN1 was assessed by western blot assay in A549 and HCC827 cells. *P < 0.05

As miRNAs can affect mRNA translation and stability by binding to complementary miRNA response elements in the 3′UTR of target mRNA, we further investigated the target mRNA of miR-326 and found that BECN3 might be the target of miR-326 in lung adenocarcinoma cells. As presented in Fig. 5A, miR-326 had the complementary binding regions on 3′ UTR of BECN1 mRNA. Besides, dual-luciferase reporter assay indicated that miR-326 mimic could decrease the luciferase activity of BECN1-3′UTR-WT group, rather than the luciferase activity of BECN1-3′UTR-MUT group, indicating the interaction between miR-326 and BECN1 (Fig. 5B, C). Furthermore, BECN1 and miR-326 were co-immunoprecipitated by Anti-Ago2 in A549 and HCC827 cells, indicating the presence of BECN1 and miR-326 in the same RISC (Fig. 5D, E). The mRNA level of BECN1 was obviously upregulated, and it was negatively correlated (*r* = − 0.7107, *P* < 0.0001) with miR-326 expression in lung adenocarcinoma tissues (*n* = 37) (Fig. 5F, G). Similarly, the protein level of BECN1 was obviously upregulated in lung adenocarcinoma tissues and cells (Fig. 5H, I). In addition, the miR-326 inhibitor increased the protein level of BECN1 in A549 and HCC827 cells (Fig. 5J). Thus, miR-128 could specifically bind BECN1 3′UTR and inhibit BECN1 expression at posttranscriptional levels.

### Overexpression of miR-326 inhibited migration, invasion, proliferation, and angiogenesis but induced apoptosis in lung adenocarcinoma cells by targeting BECN1

**Fig. 6:**
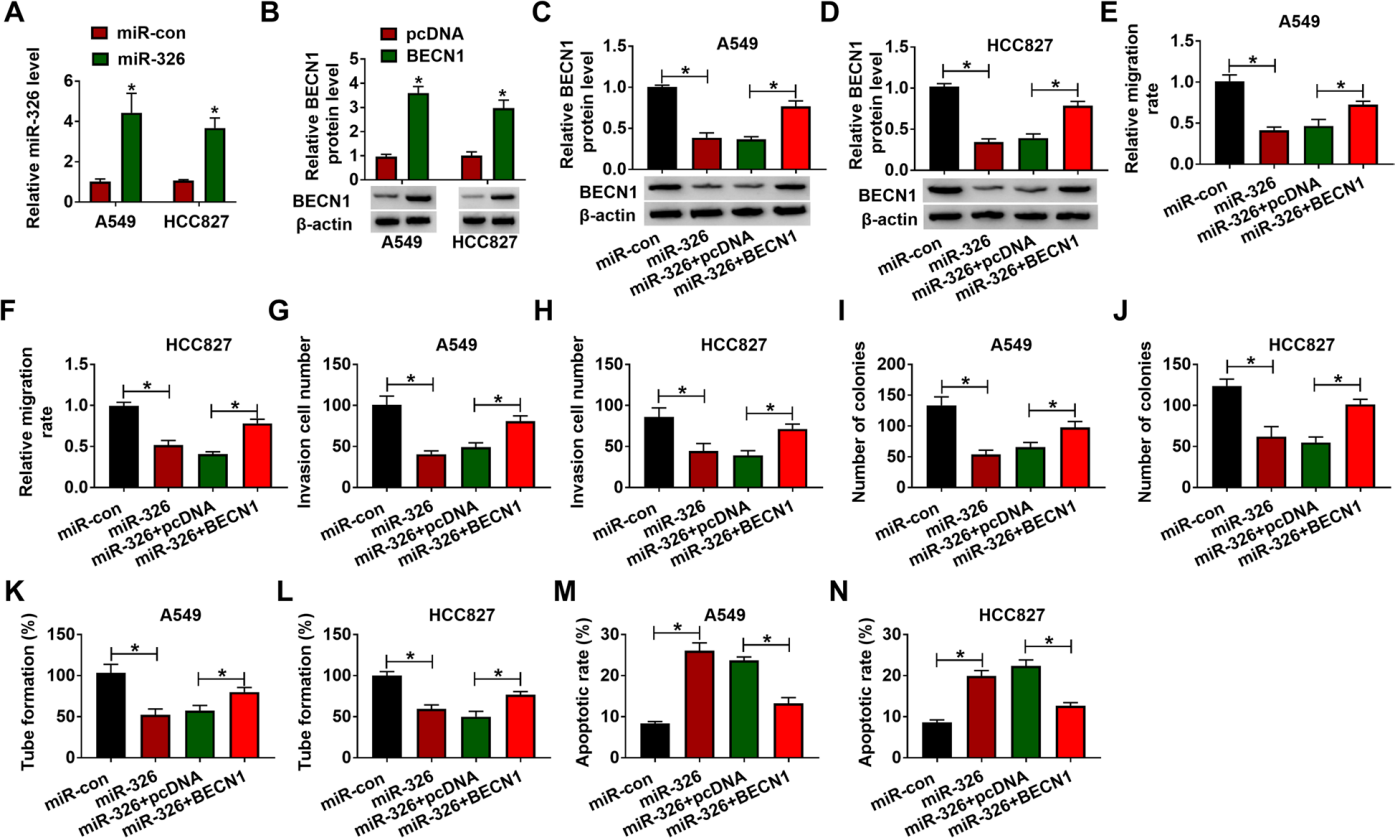
The upregulation of BECN1 reversed miR-326-induced the effect on lung adenocarcinoma cells. **A** The expression level of miR-326 was determined by RT-qPCR in A549 and HCC827 cells transfected with miR-con or miR-326. **B** The protein expression of BECN1 was quantified by western blot assay in A549 and HCC827 cells transfected with pcDNA or BECN1. **C–N** A549 and HCC827 cells were transfected with miR-con, miR-326, miR-326+pcDNA, or miR-326+BECN1. **C–D** Western blot assay was conducted in transfected A549 and HCC827 cells to assess BECN1 level. **E–H** The migration and invasion were measured by wound healing and transwell assays, respectively. **I, J** Cell proliferation was analyzed by colony-forming assay. **K, L** The tube formation was determined by matrigel angiogenesis assay. **M, N** The apoptosis was assessed by flow cytometry assay. *P < 0.05

To explore the biological function of BECN1 and miR-326 in A549 and HCC827 cells, gain-of-function assays were performed by co-transfecting miR-326 and BECN1 into A549 and HCC827 cells. As shown in Fig. 6A, B, miR-326 expression was obviously increased in miR-326 mimic-transfected A549 and HCC827 cells, while BECN1 protein level was obviously upregulated in BECN1-transfected A549 and HCC827 cells. Besides, overexpression of BECN1 could counteract miR-326-induced suppression effect on BECN1 protein level in A549 and HCC827 cells (Fig. 6C, D). Wound healing and transwell assays revealed that overexpression of miR-326 impeded cell migration and invasion in A549 and HCC827 cells, while it was overturned by upregulation of BECN1 (Fig. 6E–H, Supplementary Figure 2A). Furthermore, miR-326 mimic inhibited the colony formation and tube formation abilities of A549 and HCC827 cells, whereas these effects were partly rescued by upregulation of BECN1 (Fig. 6I–J). Flow cytometry assay uncovered that the promotion effect of miR-326 mimic on apoptosis in A549 and HCC827 cells was partly rescued by overexpression of BECN1 (Fig. 6K–N, Supplementary Figure 2B). Hence, miR-326 mediated the malignant behaviors of lung adenocarcinoma cells by regulating BECN1.

### Circ_0020850/miR-326/BECN1 axis in lung adenocarcinoma cells

**Fig. 7:**
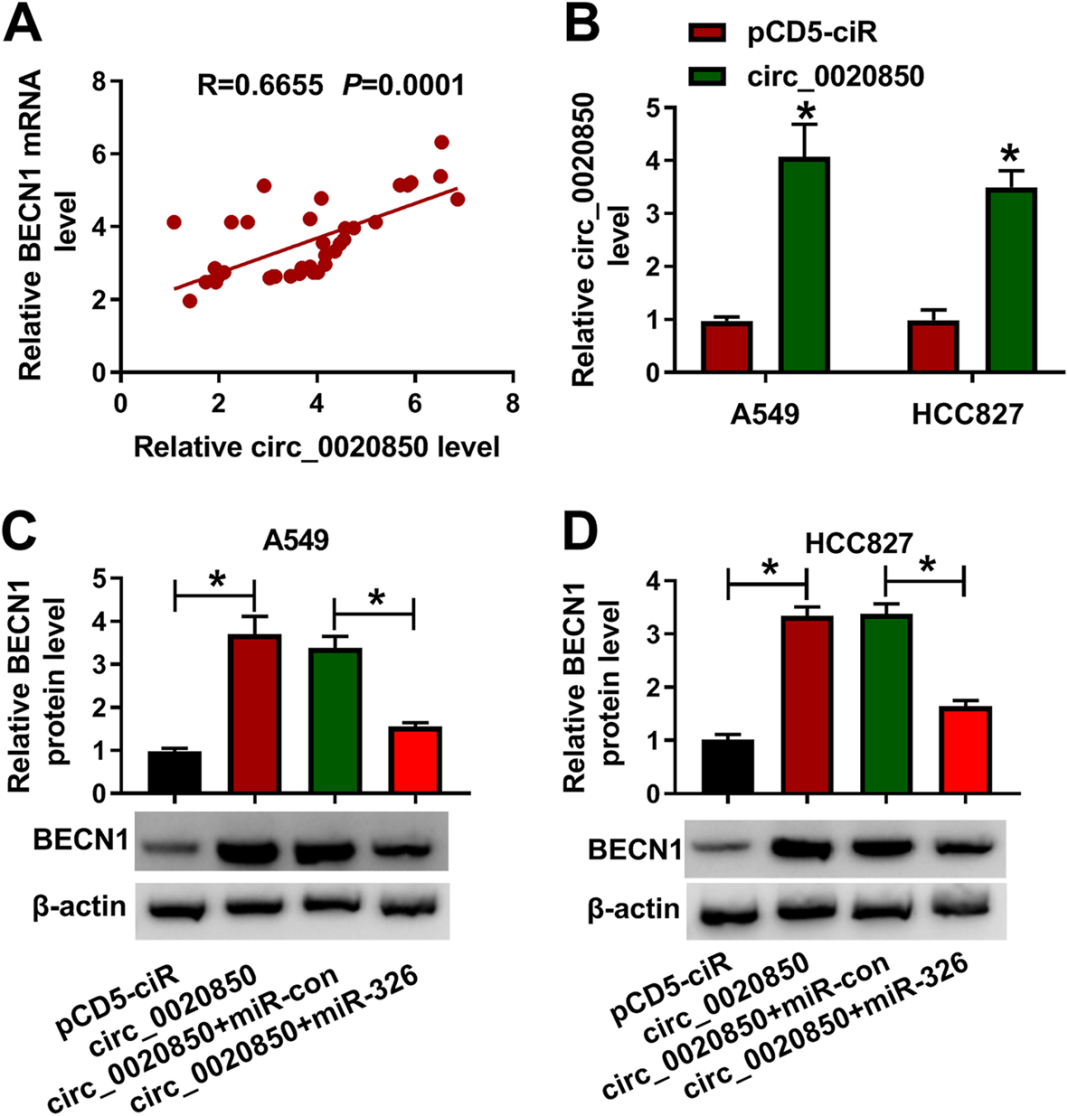
Circ_0020850 regulated BECN1 expression by targeting miR-326. **A** The correlation relationship between BECN1 and circ_0020850 was analyzed. **B** The overexpression efficiency of circ_0020850 was analyzed by RT-qPCR. **C, D** The relative expression level of BECN1 was examined by RT-qPCR and western blot assays in A549 and HCC827 cells transfected with pCD5-ciR, circ_0020850, circ_0020850+miR-con, circ_0020850+miR-326. *P < 0.05

Next, we further investigated the relationship among circ_0020850, miR-326, and BECN1 in lung adenocarcinoma cell. As shown in Fig. 7A, a positive correlation (*r* = 0.6655, *P* = 0.0001) between BECN1 and circ_0020850 was confirmed in 37 cases of lung adenocarcinoma tissues. The expression level of circ_0020850 was increased in circ_0020850-transfected A549 and HCC827 cells compared with the control group (Fig. 7B). Moreover, we found that circ_0020850 overexpression elevated the protein level of BECN1 by nearly four times, whereas this effect was partly overturned by miR-326 mimic (Fig. 7C, D). Therefore, circ_0020850 upregulated BECN1 expression by sponging miR-326 in lung adenocarcinoma cells.

### Circ_0020850 silencing impeded lung adenocarcinoma tumor growth in vivo

**Fig. 8:**
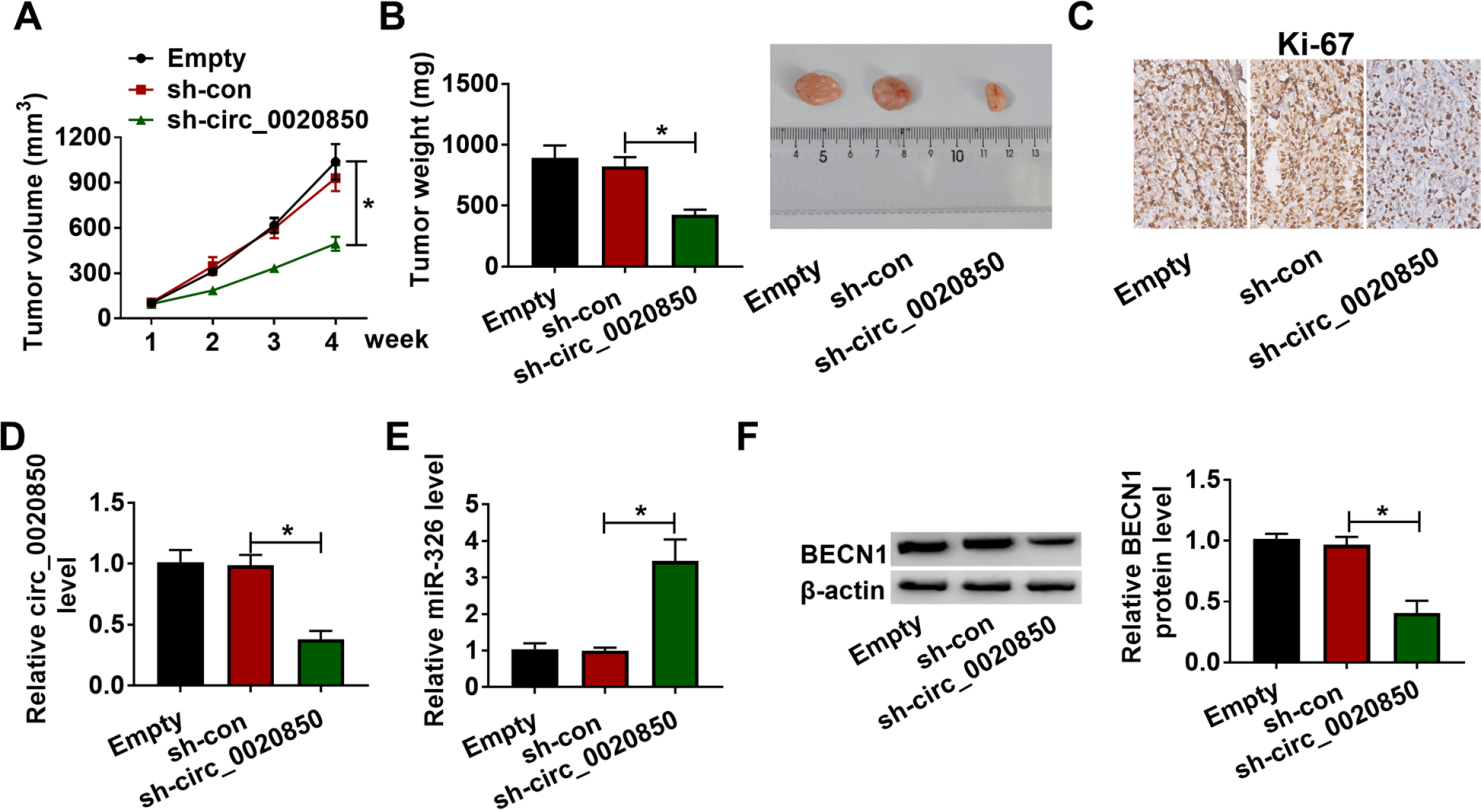
Silencing of circ_0020850 repressed tumor growth in vivo. **A, B** The growth curves and weight of xenograft tumors are presented. **C** Immunohistochemistry was performed to assess Ki-67 expression in dissected tumor tissues. **E, F** The expression levels of circ_0020850, miR-326, and BECN1 were estimated with RT-qPCR and western blot assays in dissected tumor tissues. *P < 0.05

We also investigated the in vivo function of circ_0020850 in lung adenocarcinoma. BALB/c nude mice were injected with A549 cells, A549 cells stably transfected with sh-circ_0020850 or sh-con (6 mice for each group). As exhibited in Fig. 8A, B, circ_0020850 silencing significantly suppressed the tumor volume and weight in xenograft tumor tissues in contrast with the empty group and sh-con group. IHC staining assay for Ki-67 revealed that circ_0020850 silencing significantly reduced the Ki-67-positive cells in xenograft tumor tissues, indicating that circ_0020850 silencing inhibited tumor proliferation in vivo (Fig. 8C). In addition, circ_0020850 and BECN1 expression levels were decreased while miR-326 expression level was increased in xenograft tumor tissues of sh-circ_0020850 group (Fig. 8D–F). Taken together, silencing of circ_0020850 repressed tumor growth in vivo.

## Discussion

Lung adenocarcinoma, an important histological subtype of primary lung cancer, remains a leading cause of cancer-related death. Despite progress in understanding pathophysiology in lung adenocarcinoma, its therapeutic effect is still far from satisfactory due to the lack of early diagnosis target and the high recurrence and metastasis rate of lung adenocarcinoma [5, 6]. Here, we aimed to investigate a novel biomarker for the diagnosis and treatment of lung adenocarcinoma.

CircRNAs, a novel type of noncoding RNAs, are derived from exonic or intronic sequences by precursor mRNA back-splicing [27]. Since circRNAs are more stable and abundant in body fluids (including serum exosomes, plasma, and saliva) [28], they were expected to be promising targets for the diagnosis and treatment of lung adenocarcinoma. Recently, an increasing number of studies have disclosed that circRNAs could be used as biomarkers for cancer diagnosis and treatment [29–31]. Several circRNAs including circPRKC1 [32], circPTRRA [32, 33], and circ_0001649 [34] have been confirmed to function as prognostic biomarkers for lung cancer. Thus, circRNAs have great potential to be the clinical biomarkers for the diagnosis, prognosis, and treatment of lung cancer. In current research, we found circ_0020850 was significantly upregulated in lung adenocarcinoma tissues and cells. Besides, circ_0020850 was closely related to the TNM stages, tumor size, and invasion depth of lung adenocarcinoma patients (Table 1). For survival analysis, we concluded that high circ_0020850 level was associated with low overall survival in patients, and lung adenocarcinoma patients with lymphatic metastasis had poor overall survival (Supplement Figure 3A and 3B). Functionally, circ_0020850 knockdown significantly inhibited migration, invasion, proliferation, and angiogenesis, but induced apoptosis in lung adenocarcinoma cells. Besides, circ_0020850 silencing inhibited tumor growth in vivo. These data unveiled the clinical potency of circ_0020850 as a biomarker in the diagnosis and prognosis of lung adenocarcinoma, and functional assays verified the oncogenic effects of circ_0020850, hinting that the target inhibition of circ_0020850 might be a feasible strategy for cancer therapy.

CircRNAs have been reported to function as miRNA sponges to regulate the stability of miRNAs [35]. In our research, several miRNAs were predicted to contain the complementary miRNA recognition elements for circ_0020850. RNA pull-down assay uncovered that miR-326 was the most enriched miRNA by circ_0020850 probe, indicating the strongest interaction between miR-326 and circ_0020850. And their correlation was further confirmed by dual-luciferase reporter assay and RIP assay, as the significantly reduced luciferase activity of circ_0020850-WT and miR-326 co-transfected group, as well as the enrichment of miR-326 and circ_0020850 in Ago2-related RISC. Thus, we confirmed that miR-326 was a binding target of circ_0020850. Previous research has disclosed that miR-326 was sponged by circ_0003998 and circPUM1 in lung adenocarcinoma [36, 37], and revealed the cancer-inhibitory roles of miR-326. Besides, Pan et al. presented that miR-326 exerted its functional effects on multiple cells through involvement in the downstream signaling pathways [38]. In the present research, miR-326 was lower expressed and was negatively correlated with circ_0020850 expression in lung adenocarcinoma tissues. Besides, loss-of-function experiments revealed that downregulation of miR-326 partly reversed the effects of circ_0020850 knockdown on cell migration, invasion, proliferation, angiogenesis, and apoptosis, revealing that circ_0020850 promoted the progression of lung adenocarcinoma by sponging miR-326.

MiR-326 is considered to be a tumor suppressor in several human cancers, since it could directly target downstream oncogenes, such as cyclin D1 [39], phox2a [40], and Notch1 [41]. Moreover, the overexpression of miR-326 weakened cisplatin chemoresistance of lung adenocarcinoma cells by regulating specificity protein 1 (Sp1) [42]. Similarly, Cai et al. claimed that miR-326 repressed epithelial-to-mesenchymal transition of lung adenocarcinoma cells by targeting a disintegrin and metalloprotease 17 (ADAM17) [43]. Herein, we found that BECN1 is a target of miR-326 in lung adenocarcinoma. BECN1, an essential autophagy gene, was reported to play a pivotal role in tumor biology. For example, the knockdown of BECN1 significantly enhanced breast cancer cell sensitivity to paclitaxel through caspase-dependent apoptosis [44]. Consistently, BECN1 has been reported as an oncogene in non-small-cell lung cancer, which could enhance paclitaxel resistance through involvement in autophagy in lung cancer cells [45]. However, BECN1 could repress tumorigenesis in synovial sarcoma and gastric cancer [46, 47]. The possible underlying mechanism that controversial role of BECN1 might be dependent on the cancer cell type and heterogeneity of autophagy. Additionally, Yu et al. reported that BECN1 downregulation was an independent indicator of poor prognosis of non-small-cell lung cancer patients [48]. Inconsistent with previous research, BECN1 was upregulated in lung adenocarcinoma tissues. And it functions as an oncogene in lung adenocarcinoma, as BECN1 overexpression partly reversed the suppression effect on lung adenocarcinoma progression. In addition, we found that circ_0020850 could upregulate BECN1 expression by sponging miR-326 in lung adenocarcinoma. However, whether circ_0020850/miR-326/BECN1 axis mediated the progression of lung adenocarcinoma by regulating autophagy has not been explored. In the current research, our results partially disclosed that circ_0020850 promotes the malignant behaviors of lung adenocarcinoma by regulating miR-326/BECN1 axis.

## Conclusion

In summary, circ_0020850 knockdown inhibited migration, invasion, proliferation, and angiogenesis but stimulated apoptosis in lung adenocarcinoma cells, which might be dependent on miR-326/BECN1 networks and ceRNA mechanisms.

## Supplementary Information

**Additional file 1 **: **Supplement Figure 1**. Knockdown of circ_0020850-induced effects on lung adenocarcinoma cells were abolished by inhibition of miR-326. (A-B) The representative images of the transwell and flow cytometry assays were presented in A549 and HCC827 cells transfected with si-con, si-circ_0020850, si-circ_0020850+in-miR-con, or si-circ_0020850+in-miR-326.

**Additional file 2 **: **Supplement Figure 2**. The upregulation of BECN1 reversed miR-326-induced the effect on lung adenocarcinoma cells**.** (A-B) The representative images of the transwell and flow cytometry assays were presented in A549 and HCC827 cells transfected with miR-con, miR-326, miR-326+pcDNA, or miR-326+BECN1.

**Additional file 3 **: **Supplement Figure 3**. Overall survival linked to circ_0020850 level and lymphatic metastasis. (A) Kaplan-Meier analysis revealed the effect of circ_0020850 level on overall survival (*P*=0.0305). (B) Kaplan-Meier analysis revealed the effect of lymphatic metastasis on overall survival (*P*=0.0131).

**Additional file 4.**

## Data Availability

The data sets used and/or analyzed during the current study are available from the corresponding author on reasonable request.
