# Supplementary figures and images for: Hsa_circ_0020850 promotes the malignant behaviors of lung adenocarcinoma by regulating miR-326/BECN1 axis
Source: World J Surg Oncol. 2022 Jan 10;20:13. doi: 10.1186/s12957-021-02480-3 (PMC8750879; doi:10.1186/s12957-021-02480-3)

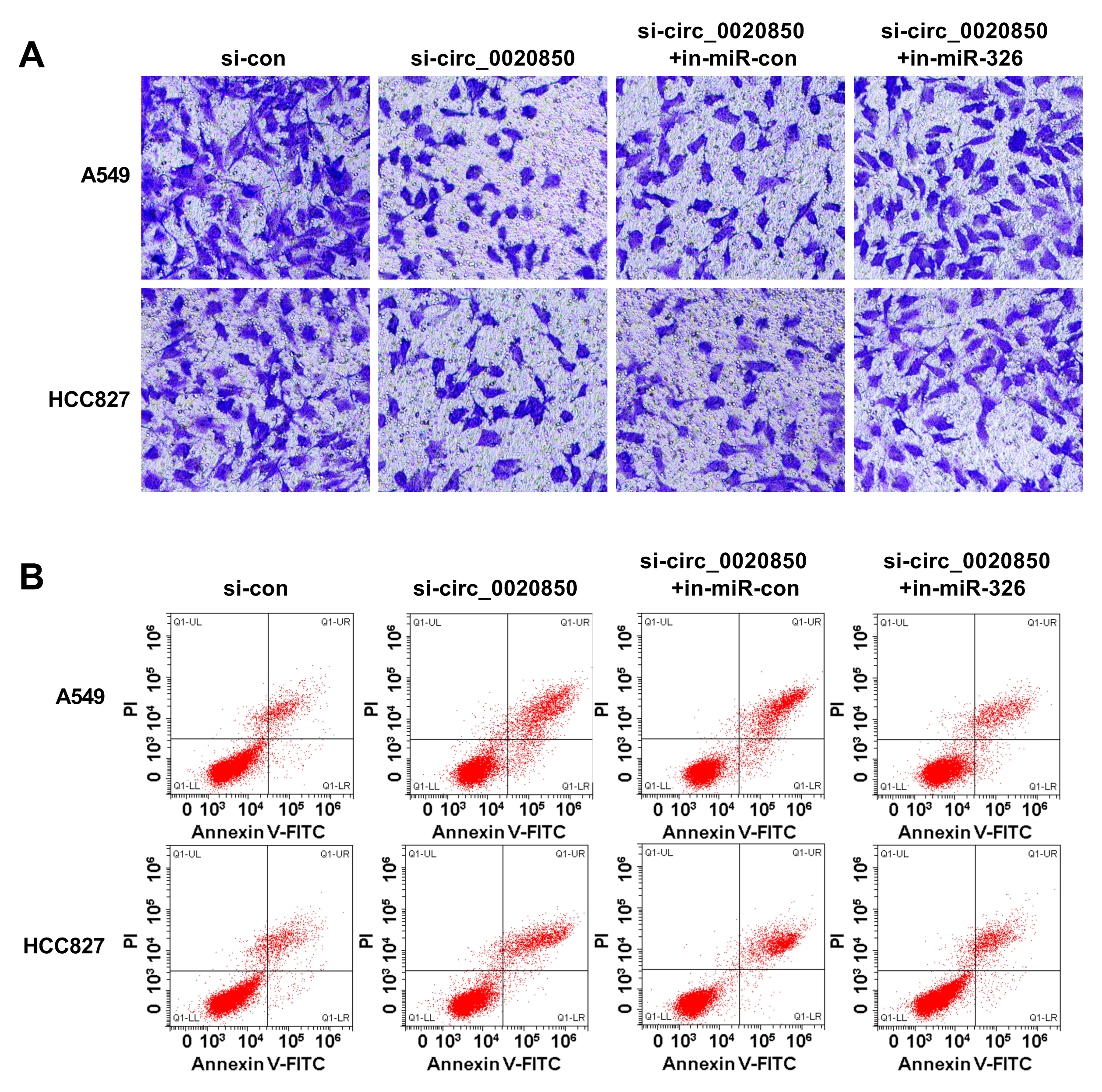

Supplement: Supplementary file 1 — Additional file 1 : Supplement Figure 1. Knockdown of circ_0020850-induced effects on lung adenocarcinoma cells were abolished by inhibition of miR-326. (A-B) The representative images of the transwell and flow cytometry assays were presented in A549 and HCC827 cells transfected with si-con, si-circ_0020850, si-circ_0020850+in-miR-con, or si-circ_0020850+in-miR-326. [file 12957_2021_2480_MOESM1_ESM.tif]

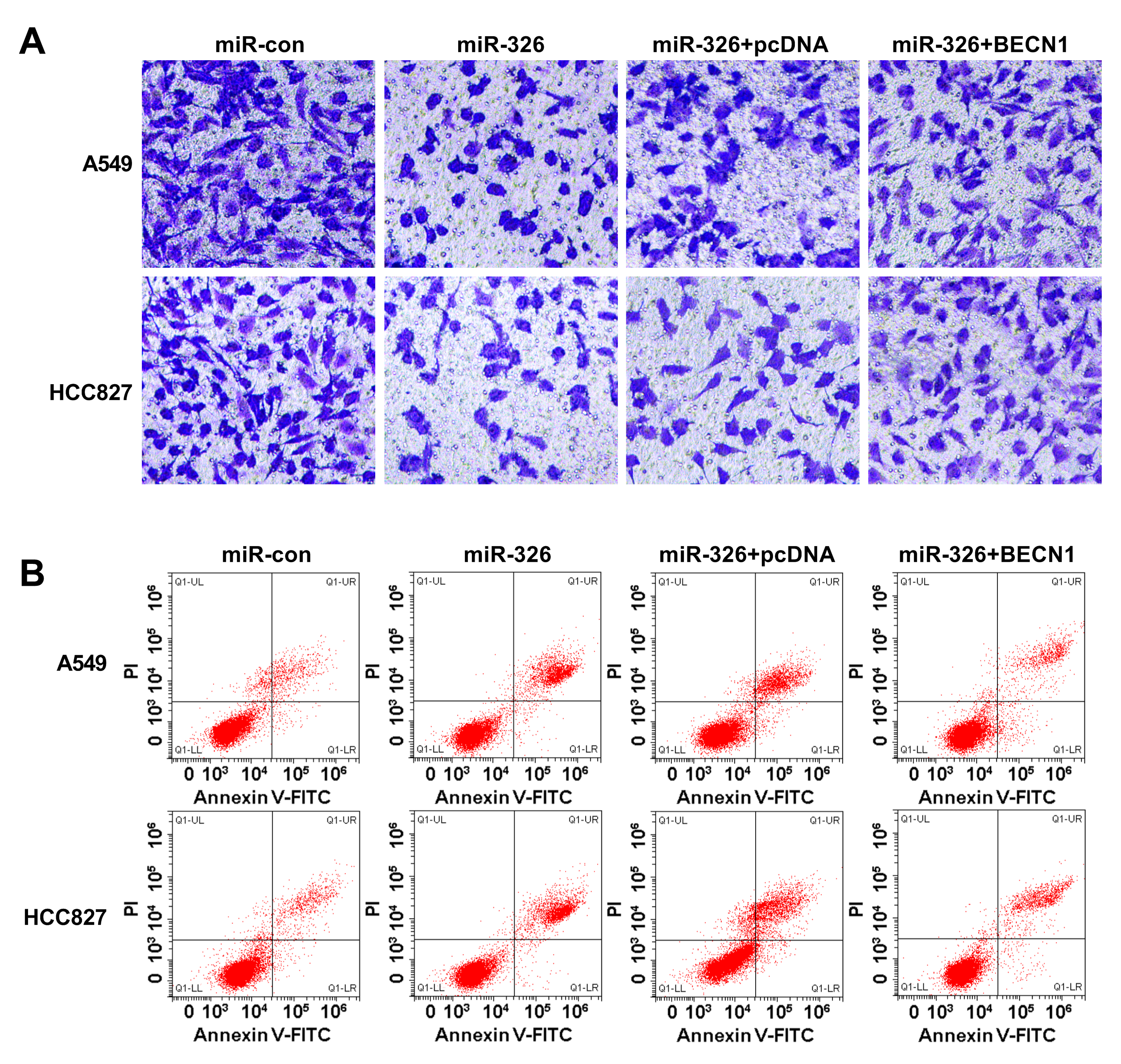

Supplement: Supplementary file 2 — Additional file 2 : Supplement Figure 2. The upregulation of BECN1 reversed miR-326-induced the effect on lung adenocarcinoma cells. (A-B) The representative images of the transwell and flow cytometry assays were presented in A549 and HCC827 cells transfected with miR-con, miR-326, miR-326+pcDNA, or miR-326+BECN1. [file 12957_2021_2480_MOESM2_ESM.tif]

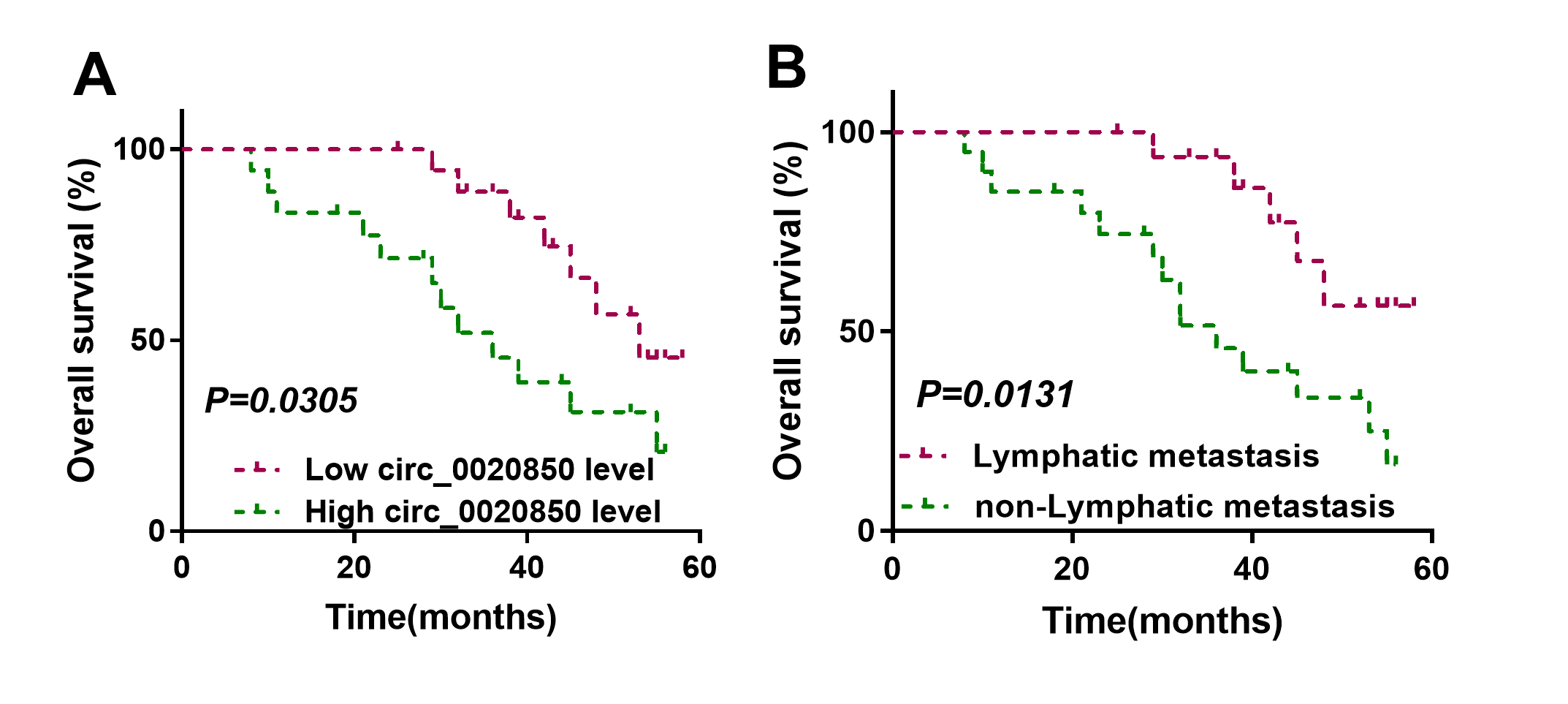

Supplement: Supplementary file 3 — Additional file 3 : Supplement Figure 3. Overall survival linked to circ_0020850 level and lymphatic metastasis. (A) Kaplan-Meier analysis revealed the effect of circ_0020850 level on overall survival (P=0.0305). (B) Kaplan-Meier analysis revealed the effect of lymphatic metastasis on overall survival (P=0.0131). [file 12957_2021_2480_MOESM3_ESM.tif]
