# Supplementary material for: Hsa_circ_0020850 promotes the malignant behaviors of lung adenocarcinoma by regulating miR-326/BECN1 axis
Source: World J Surg Oncol. 2022 Jan 10;20:13. doi: 10.1186/s12957-021-02480-3 (PMC8750879; doi:10.1186/s12957-021-02480-3)

5H-β-actin
42KD

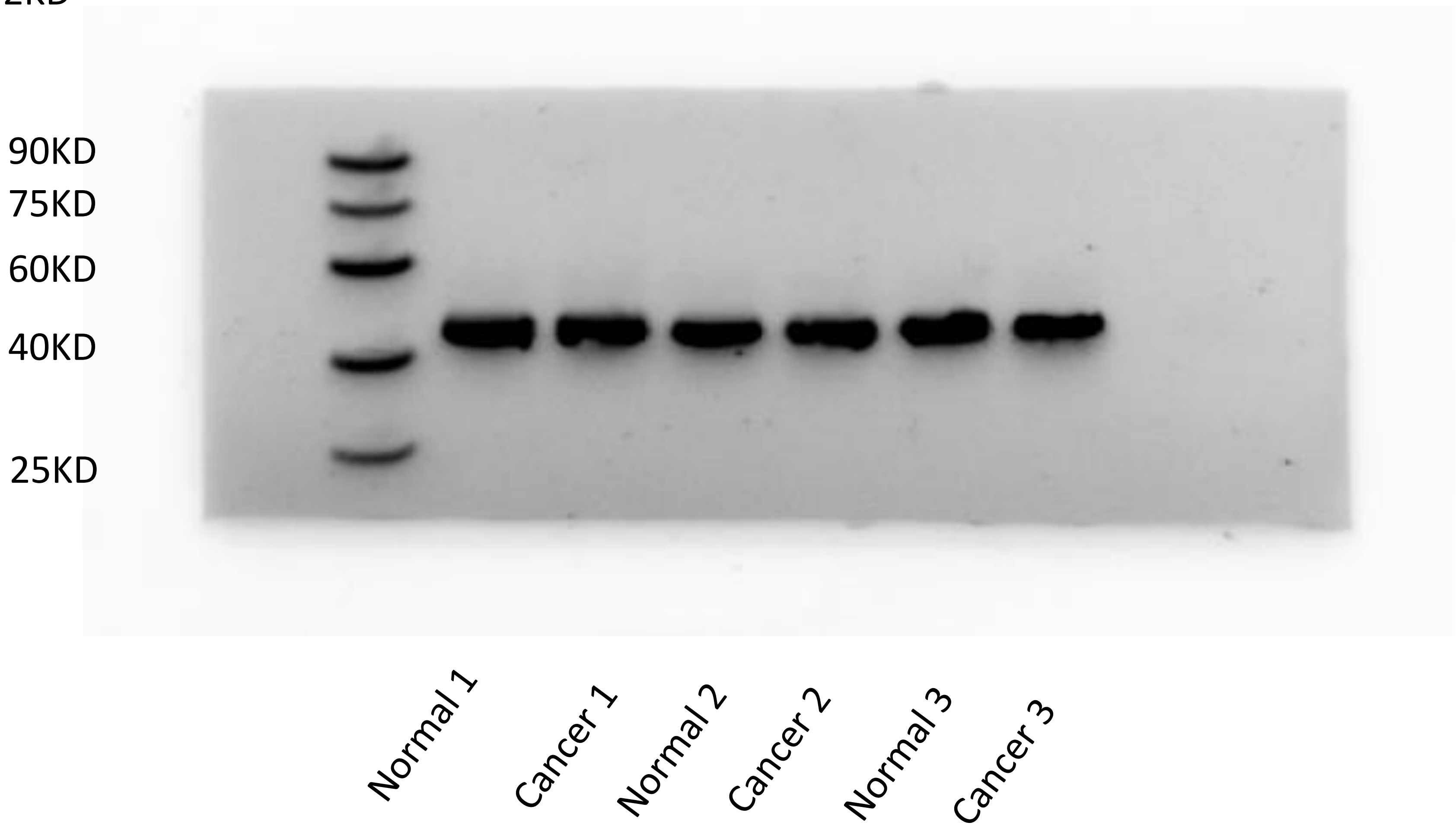

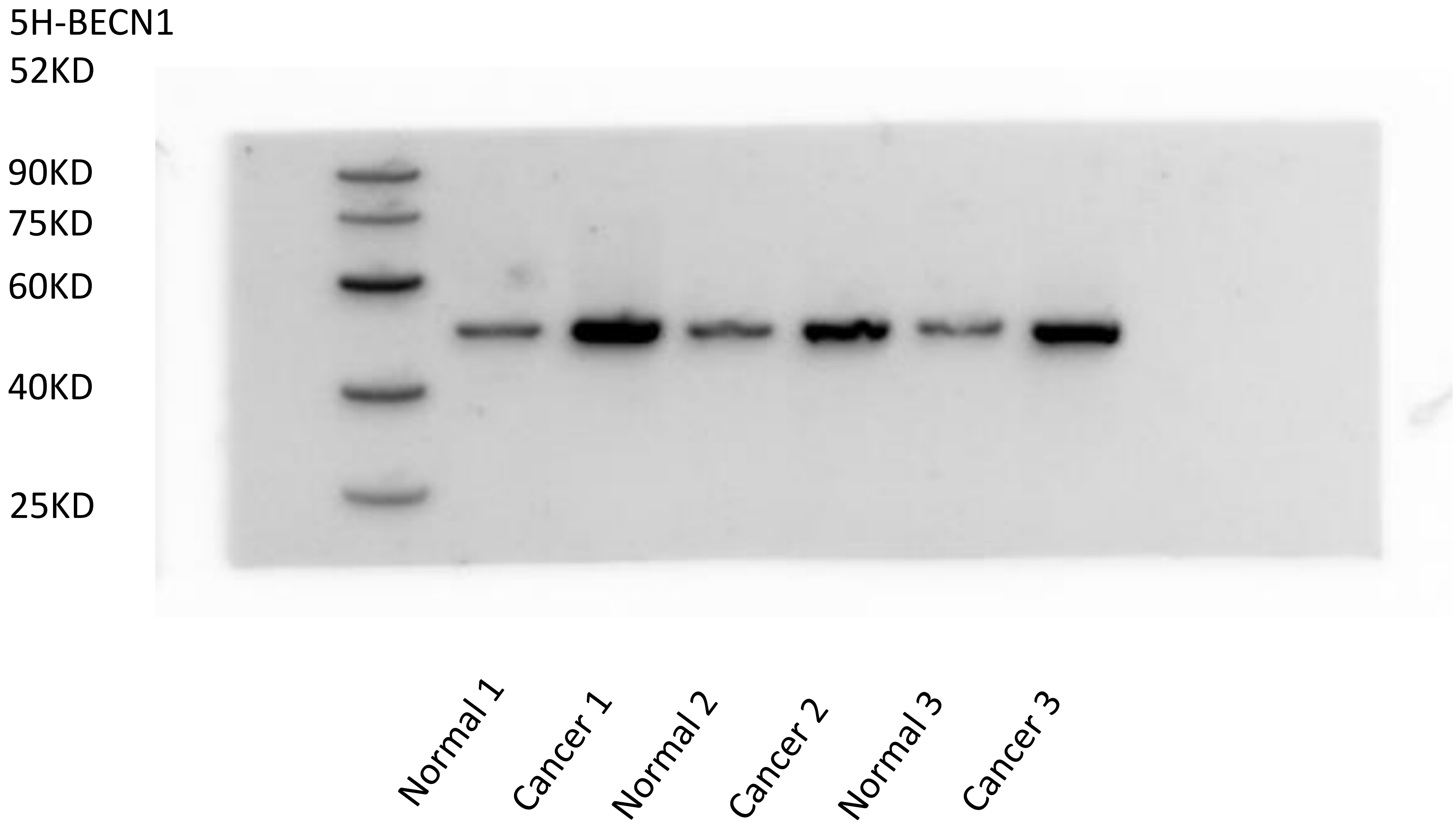
5H-BECN1
52KD
90KD
75KD
60KD
40KD
25KD
Normal 1
Cancer 1
Normal 2
Cancer 2
Normal 3
Cancer 3

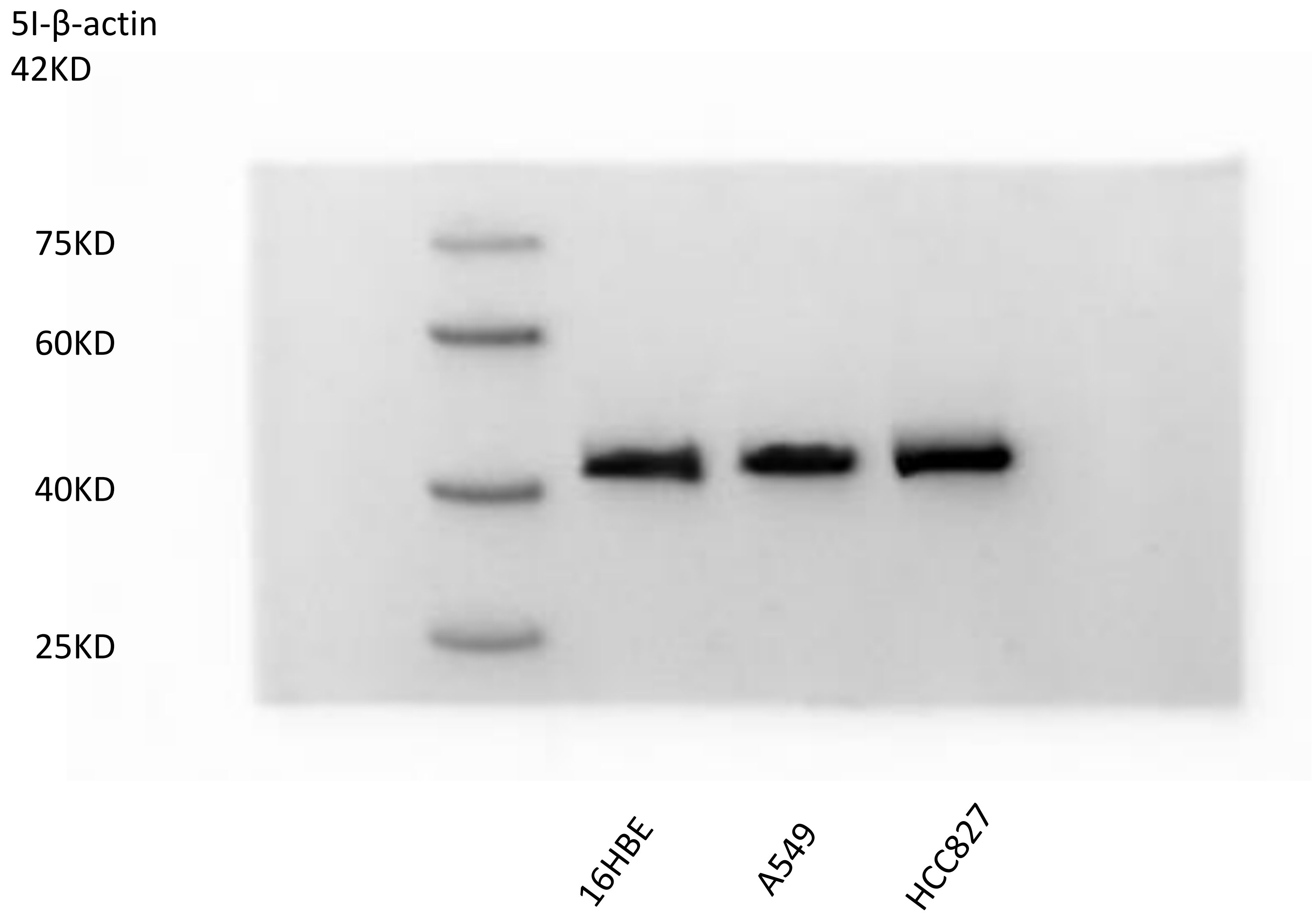
5I-β-actin
42KD
75KD
60KD
40KD
25KD
16HBE
A549
HCC827

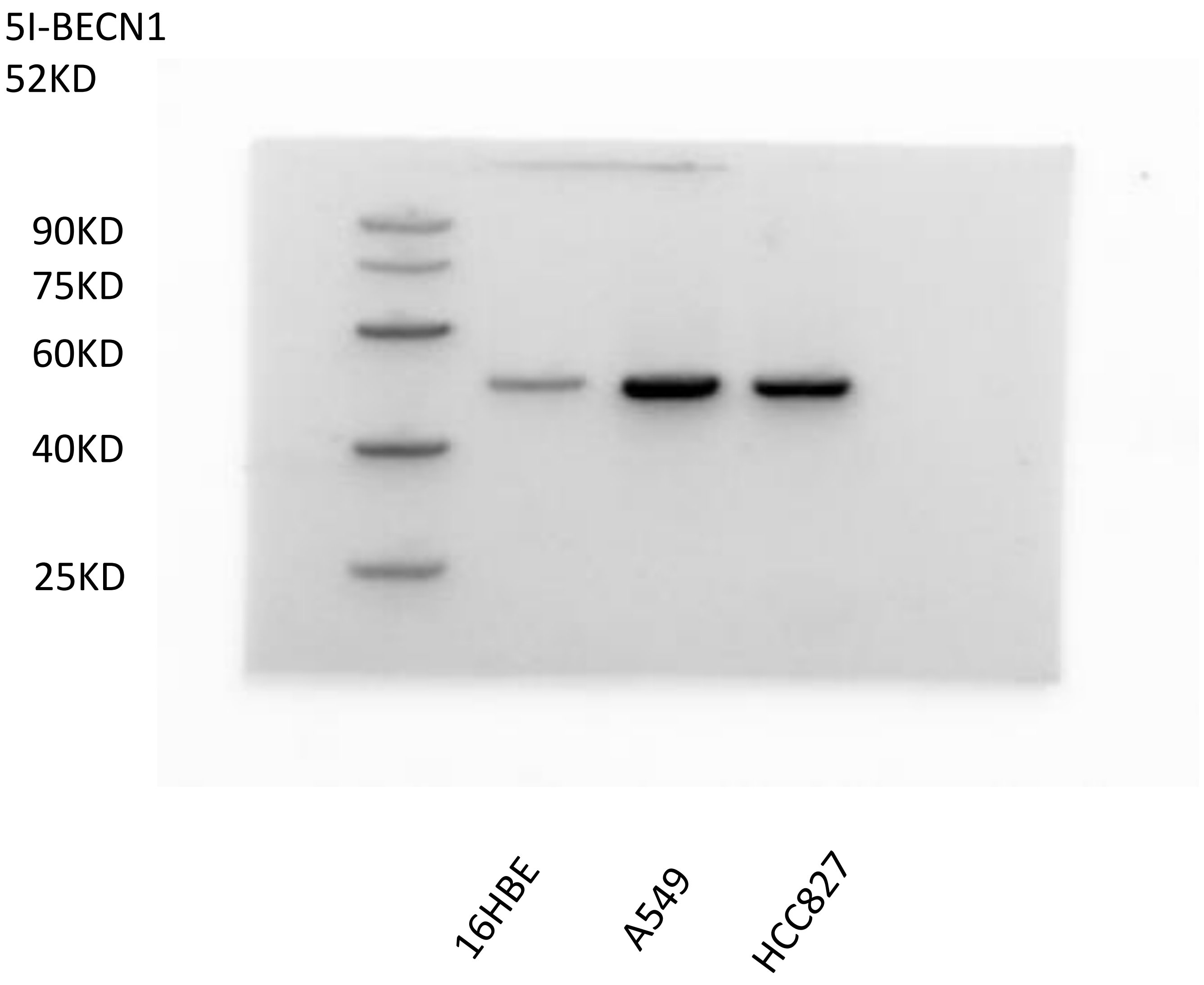
5I-BECN1
52KD
90KD
75KD
60KD
40KD
25KD
16HBE
A549
HCC827

5J-β-actin
42KD
A549

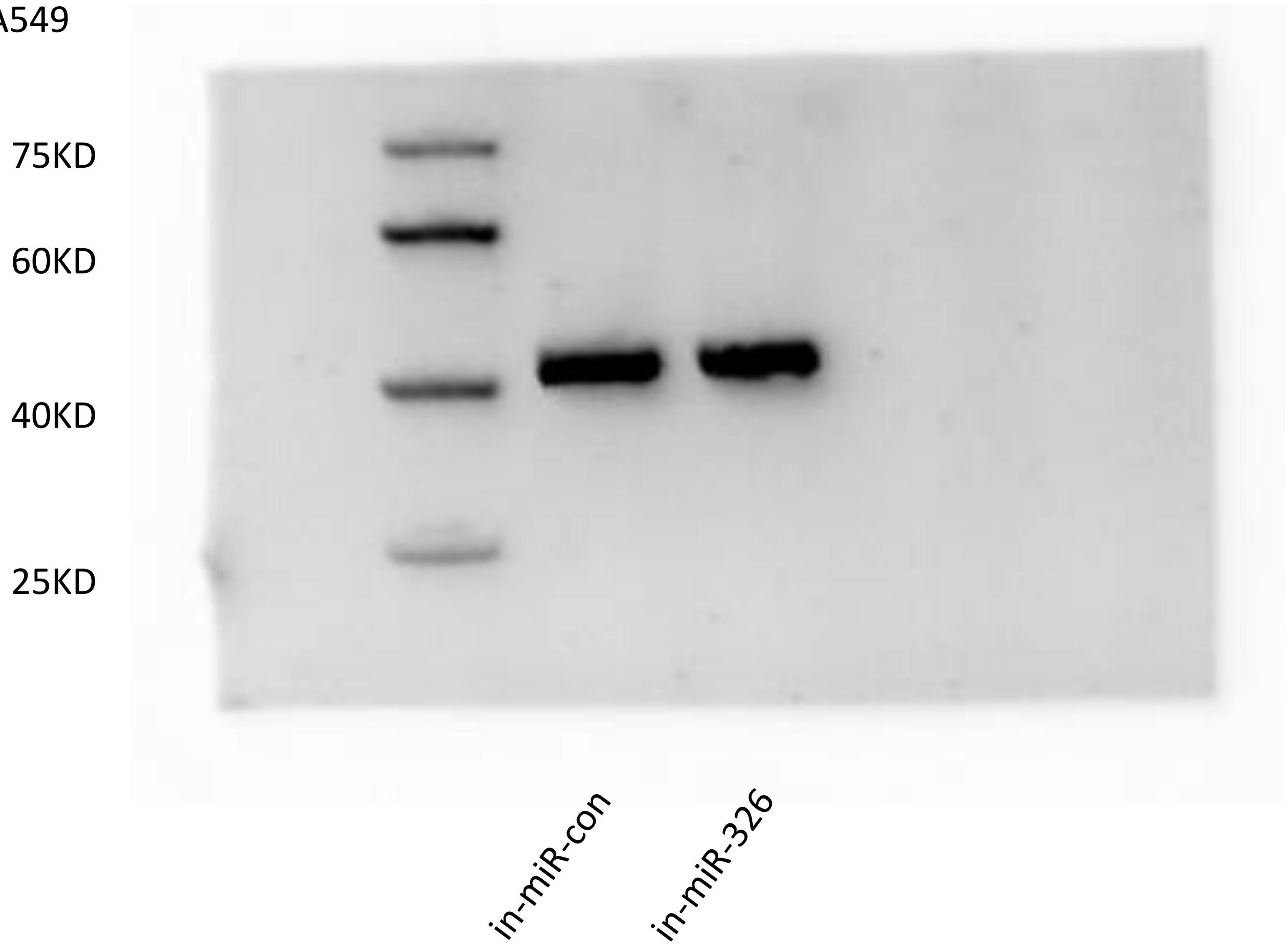

5J-BECN1
52KD
A549

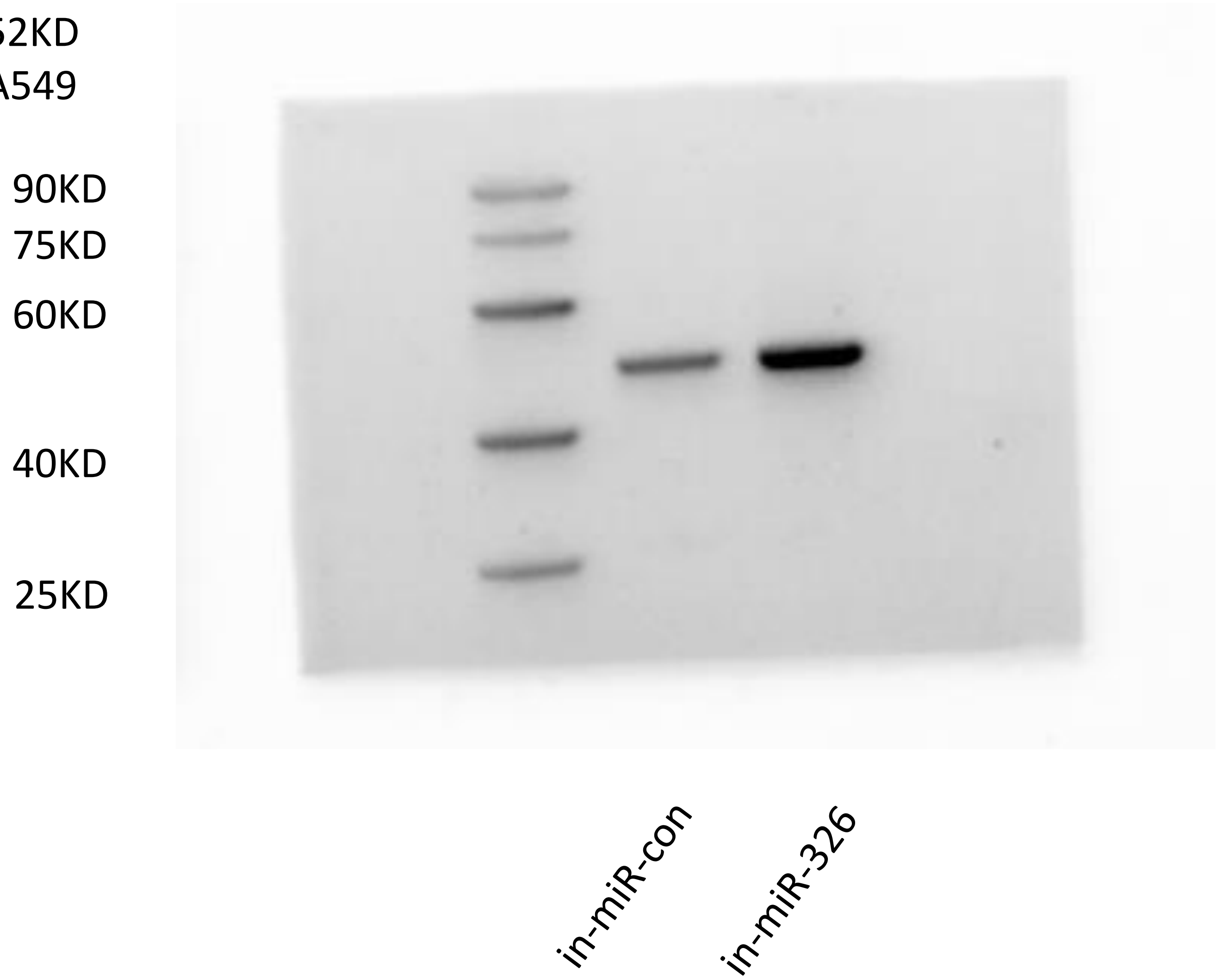

5J-β-actin
42KD
HCC827

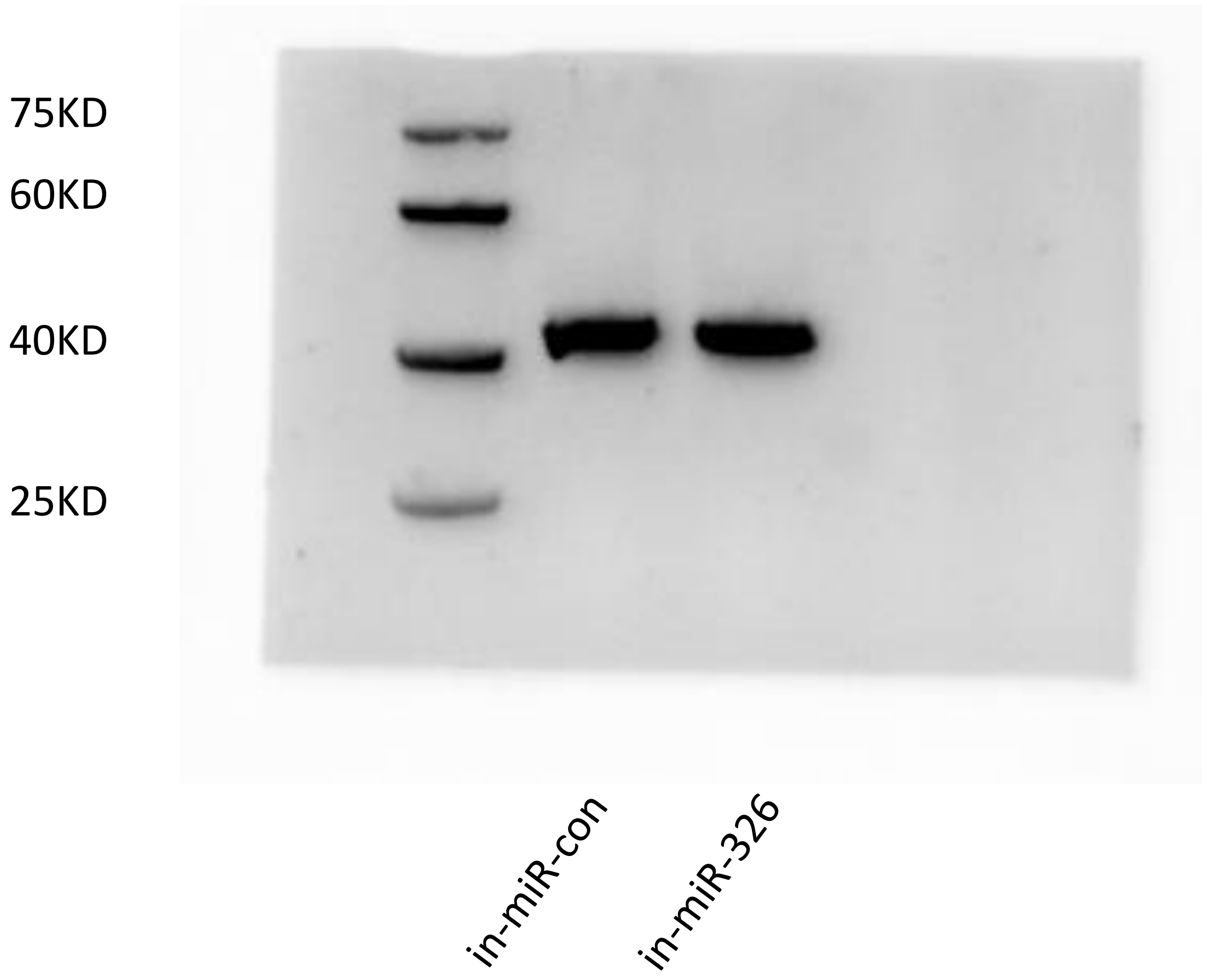

5J-BECN1
52KD
HCC827

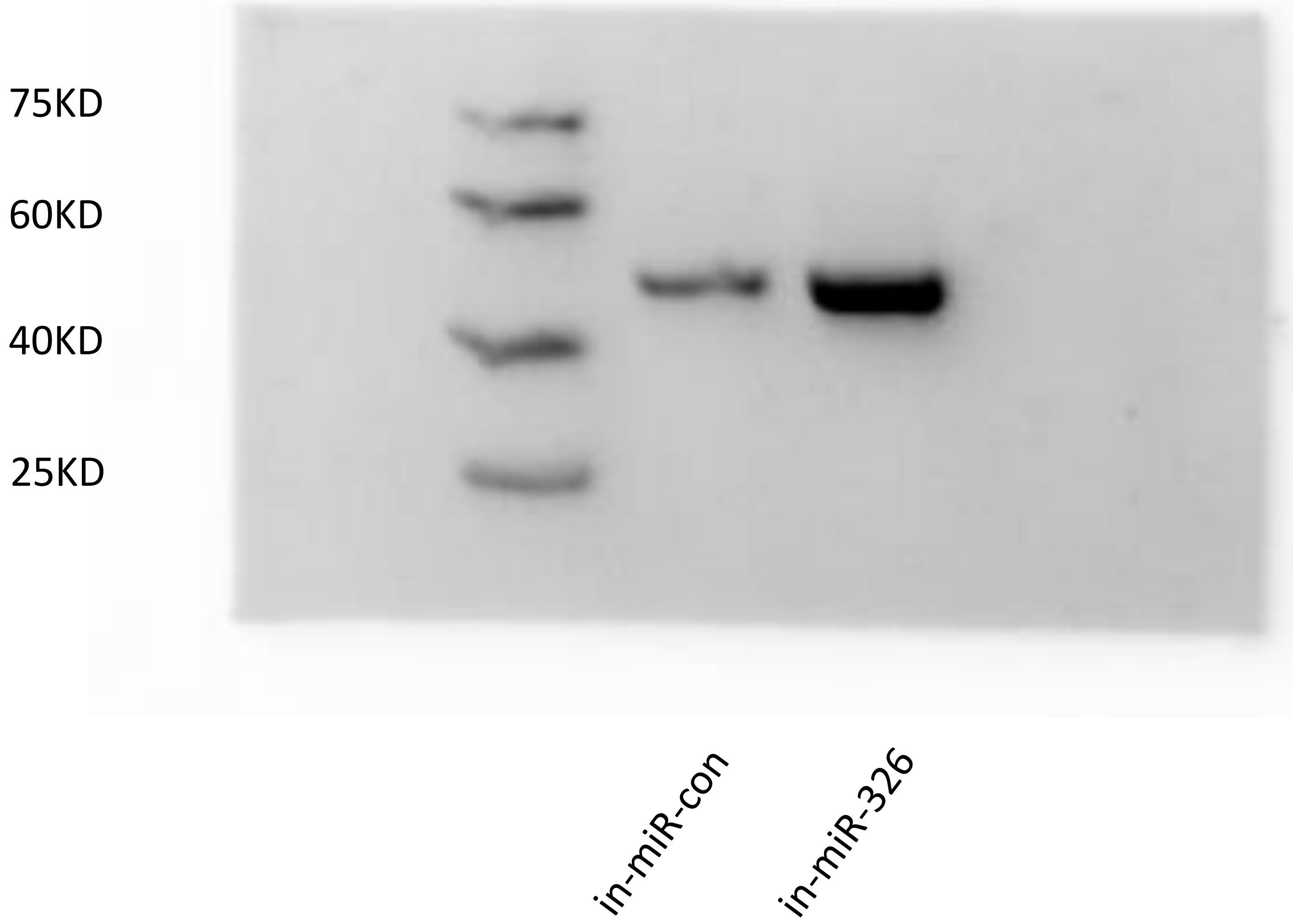

6B-β-actin
42KD
A549

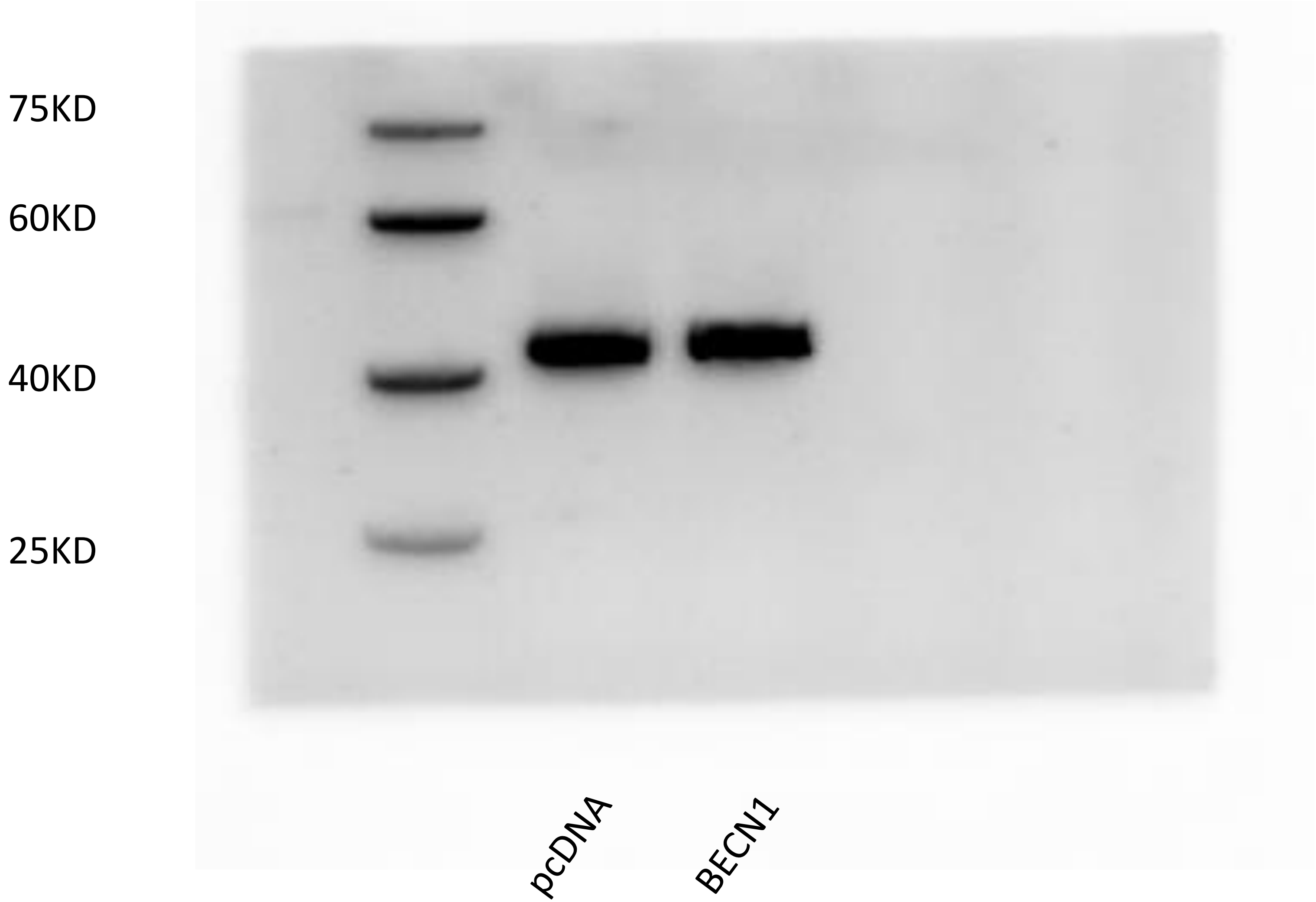

6B-BECN1
52KD
A549

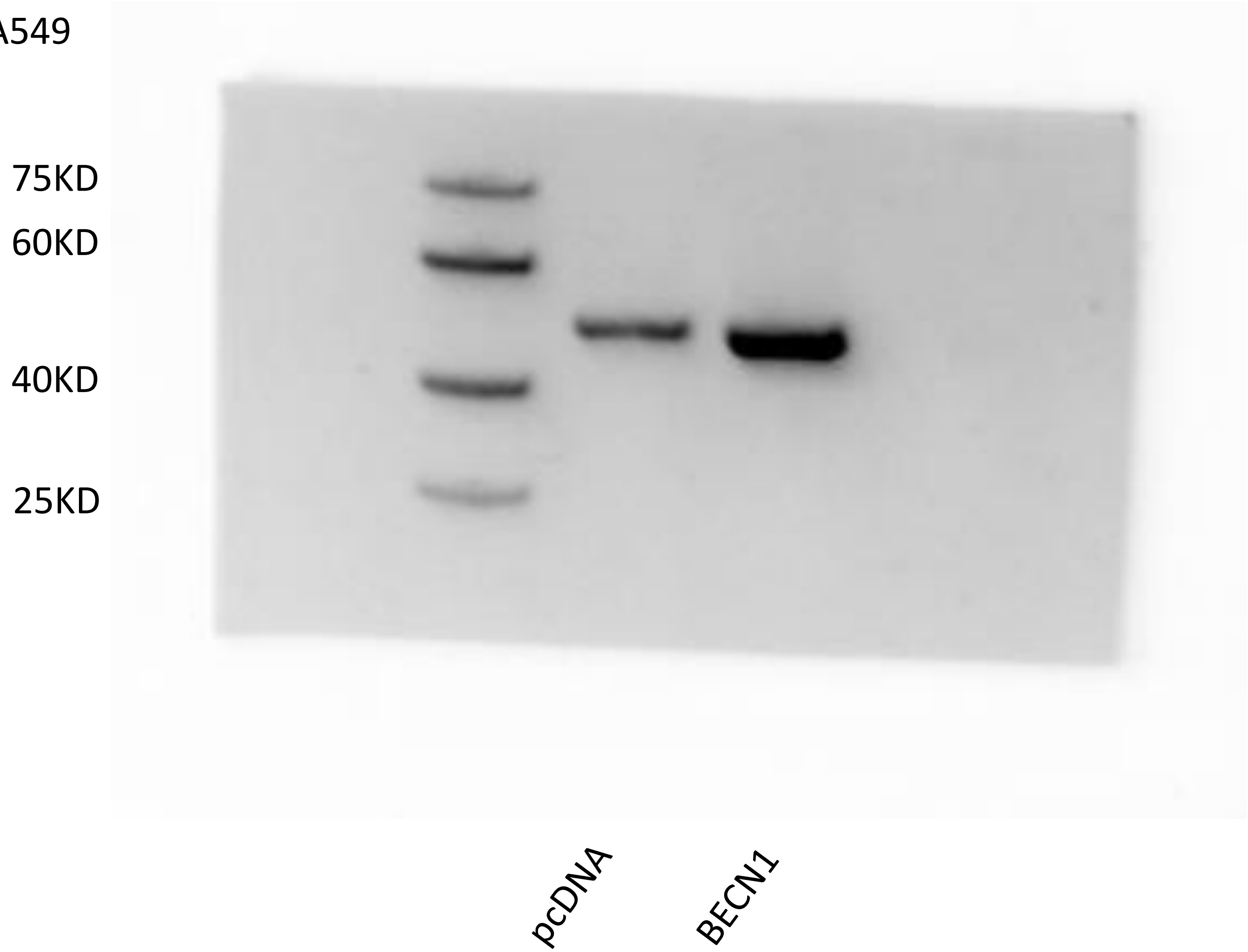

6B-β-actin
42KD
HCC827

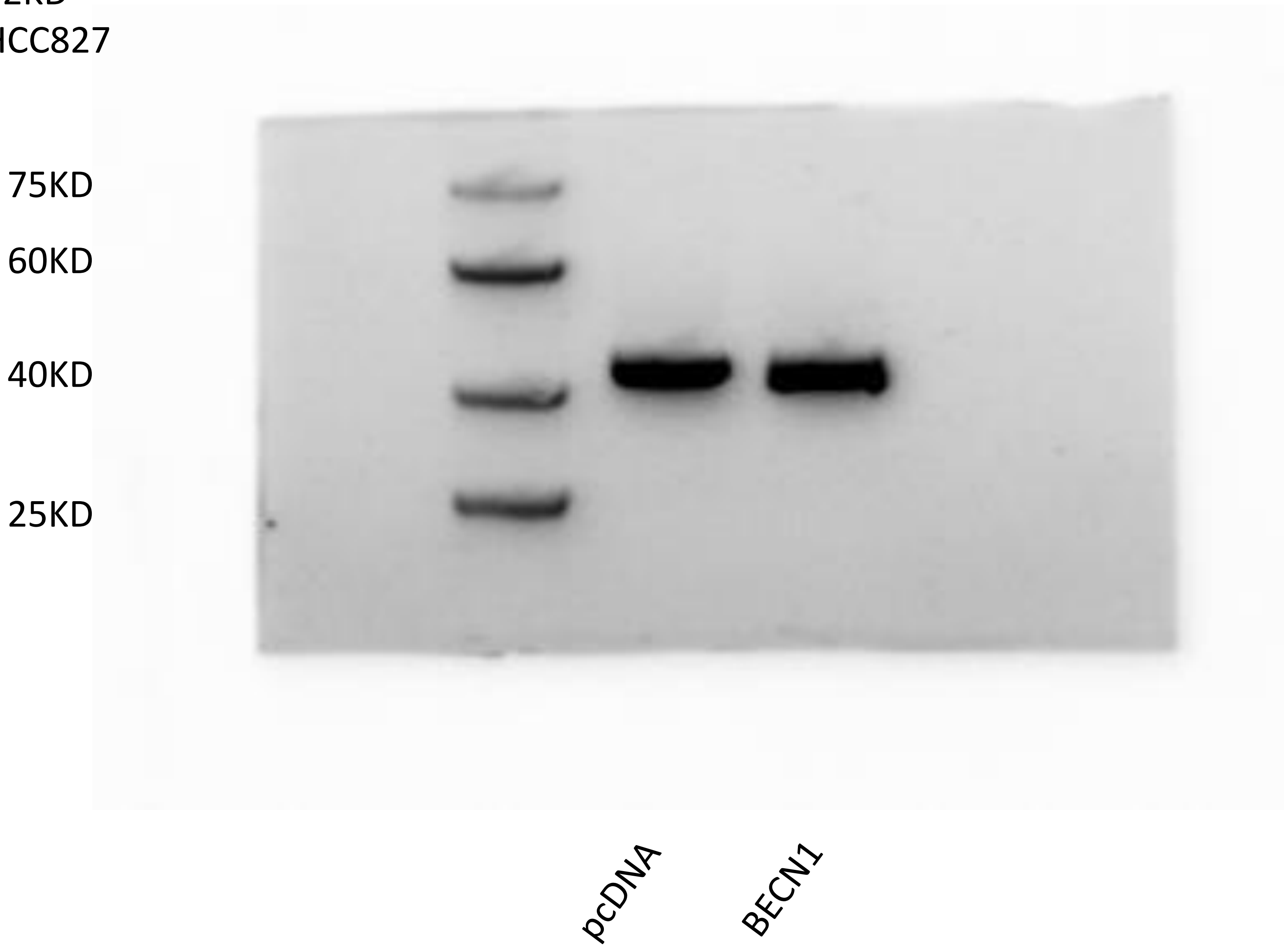

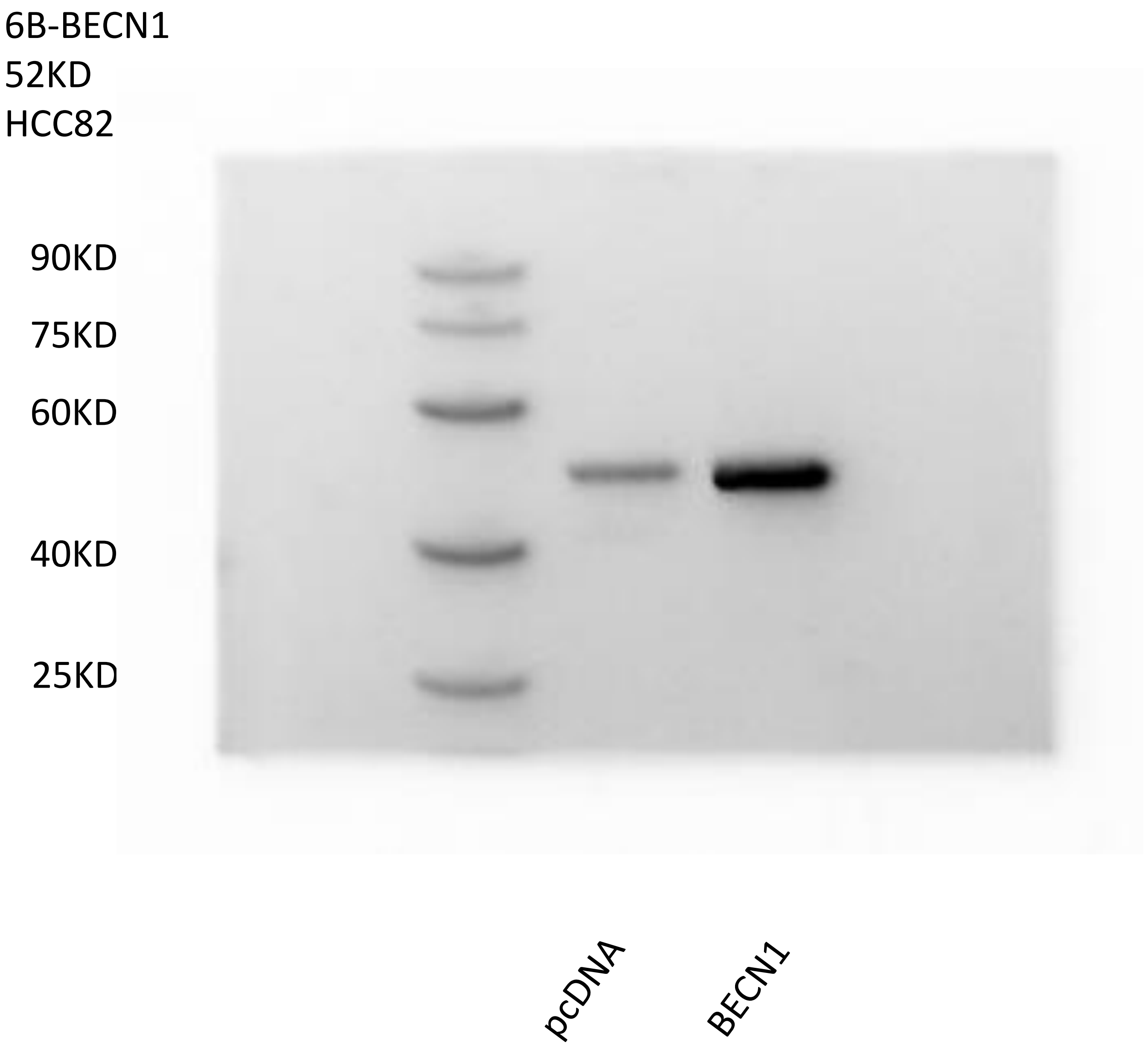
6B-BECN1
52KD
HCC82
90KD
75KD
60KD
40KD
25KD
pcDNA
BECN1

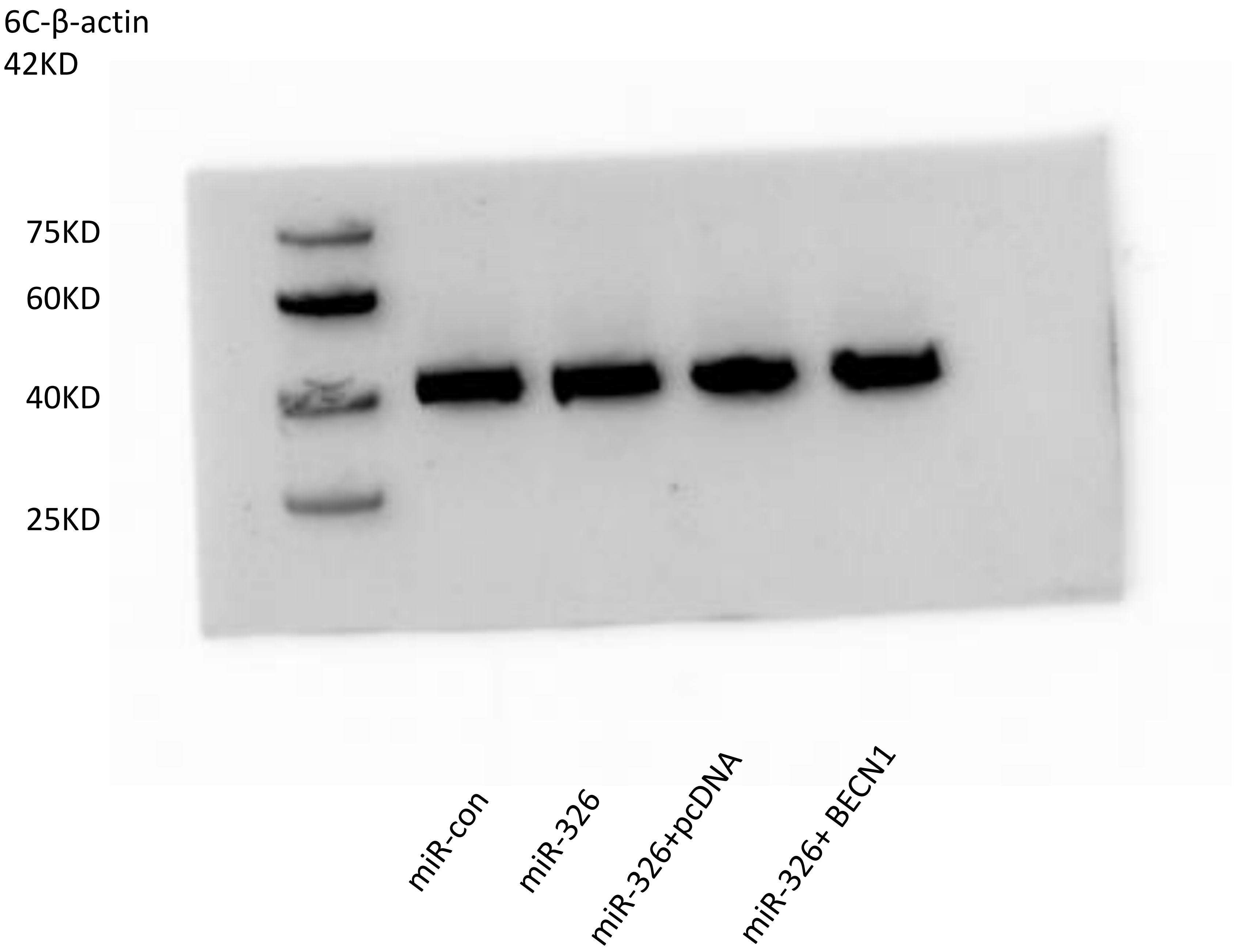
6C-β-actin
42KD
75KD
60KD
40KD
25KD
miR-con
miR-326
miR-326+pcDNA
miR-326+ BECN1

6C-BECN1
52KD

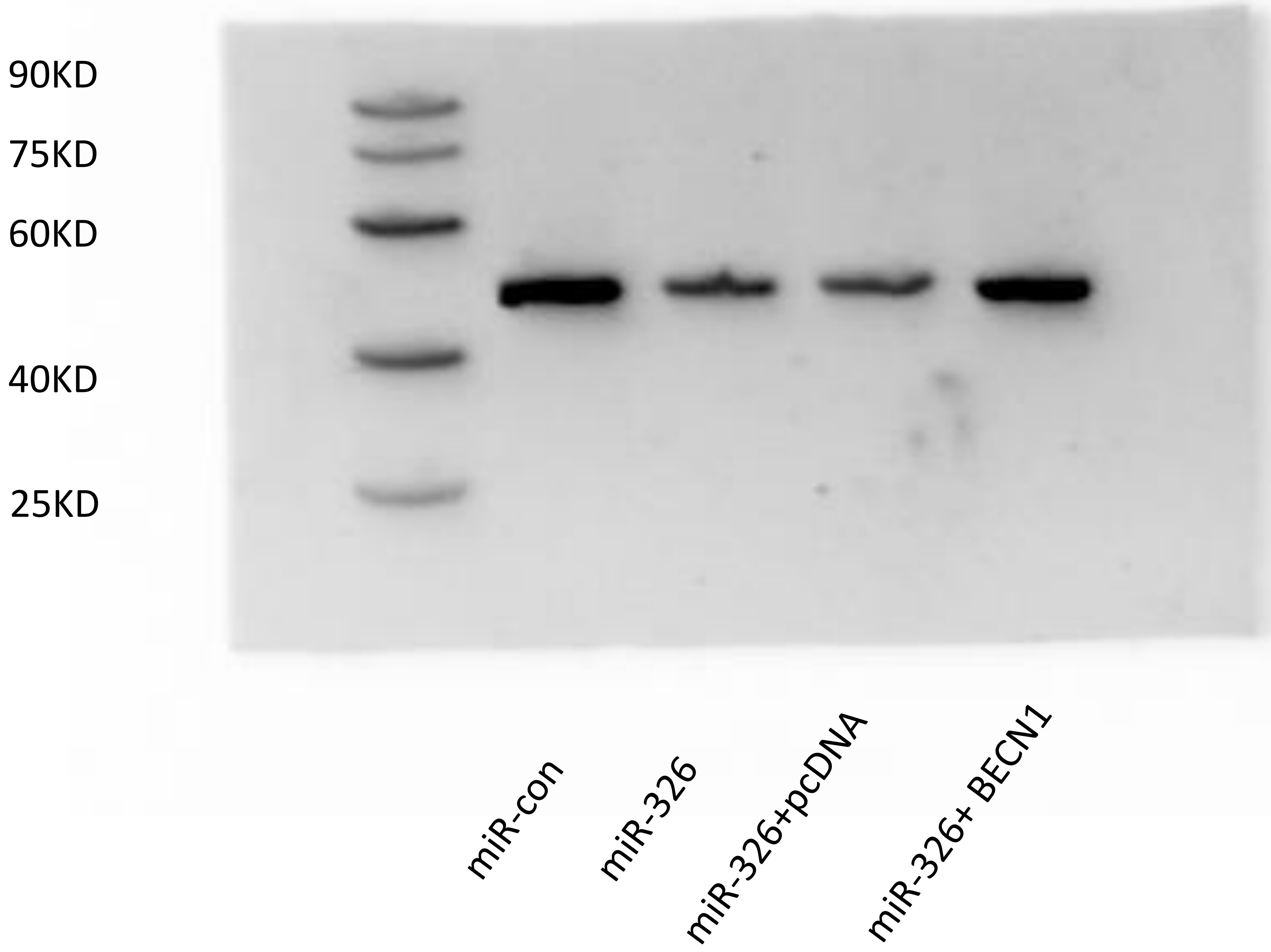

6D-β-actin
42KD

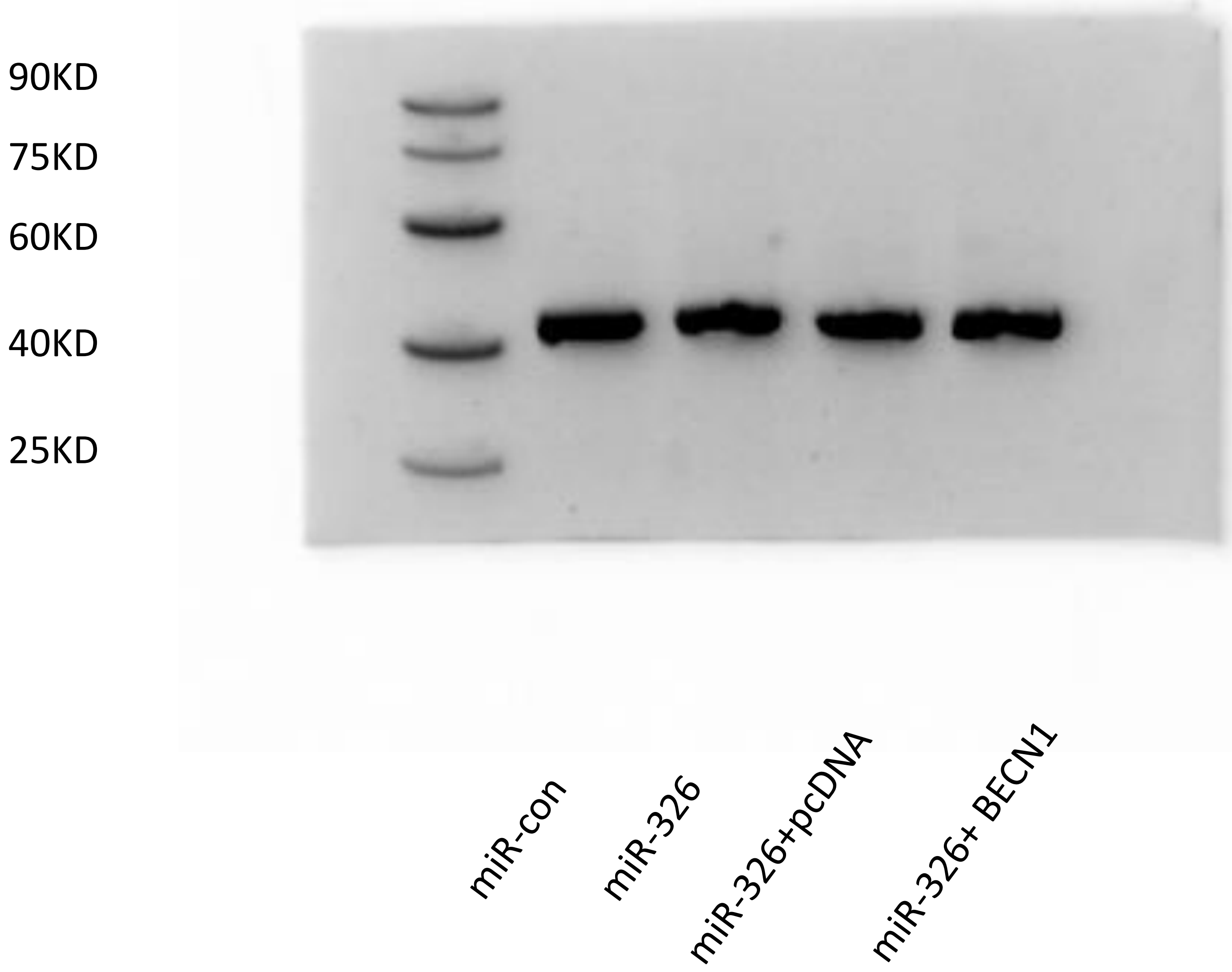

6D-BECN1
52KD

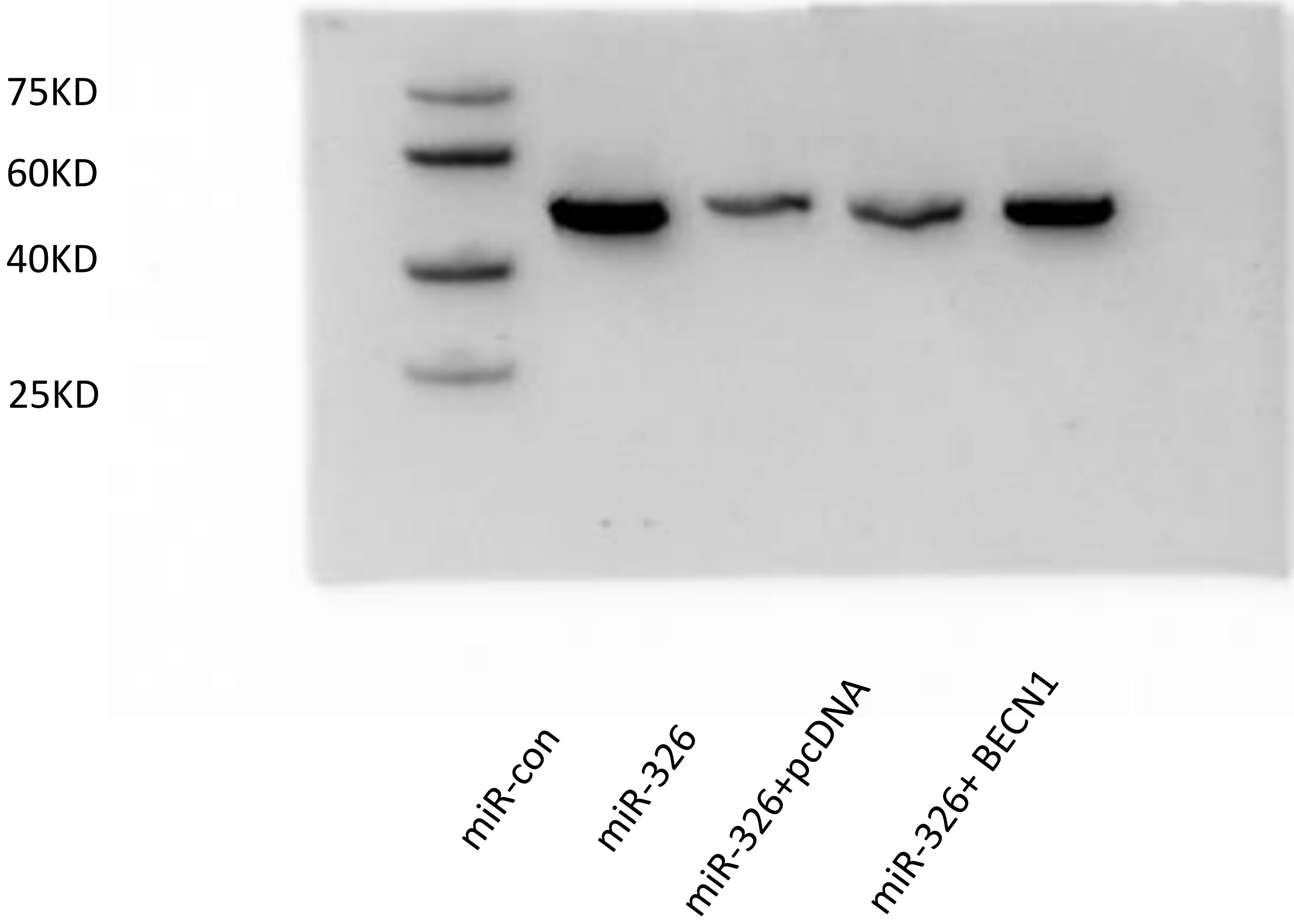

7C-β-actin
42KD

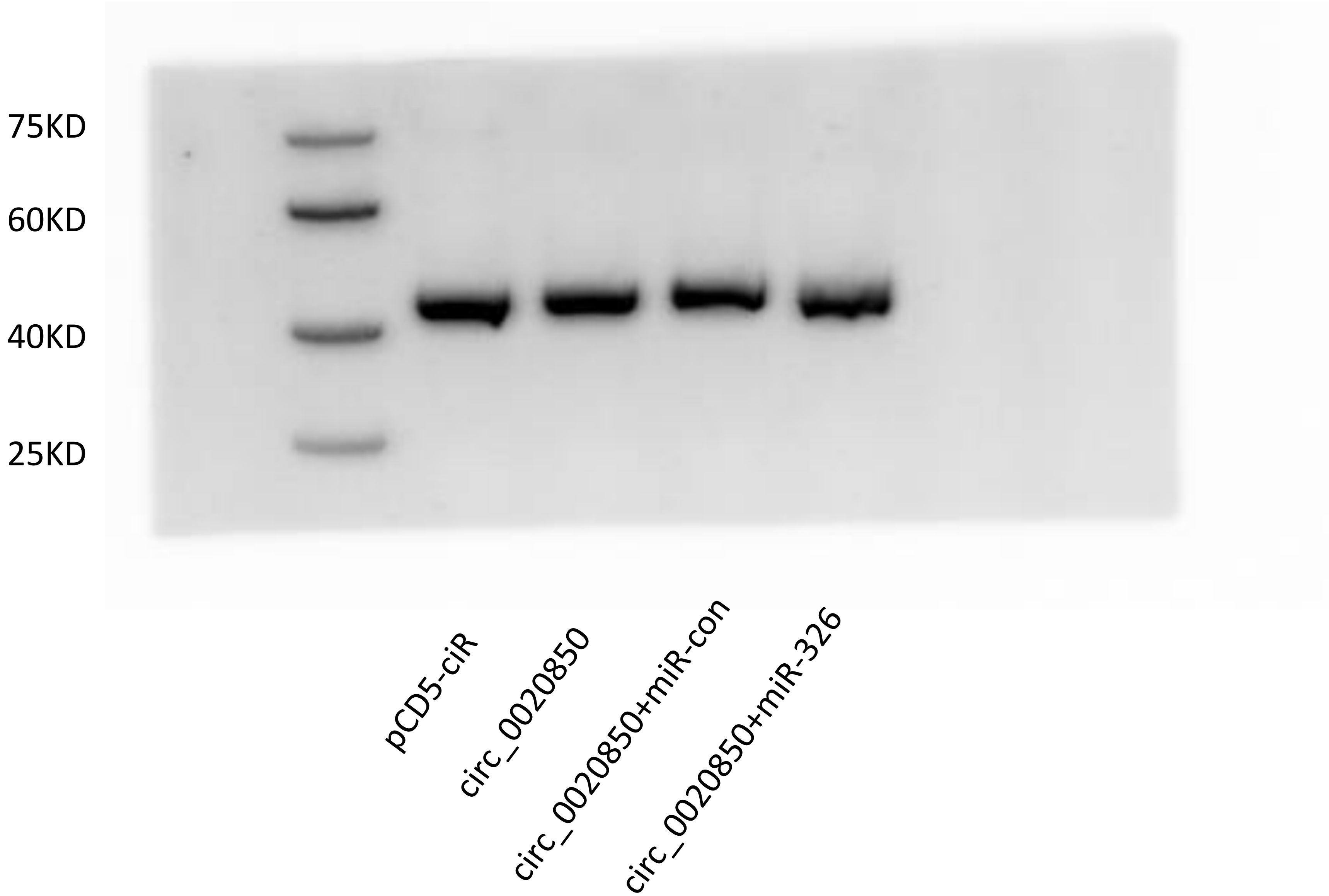

7C-BECN1
52KD

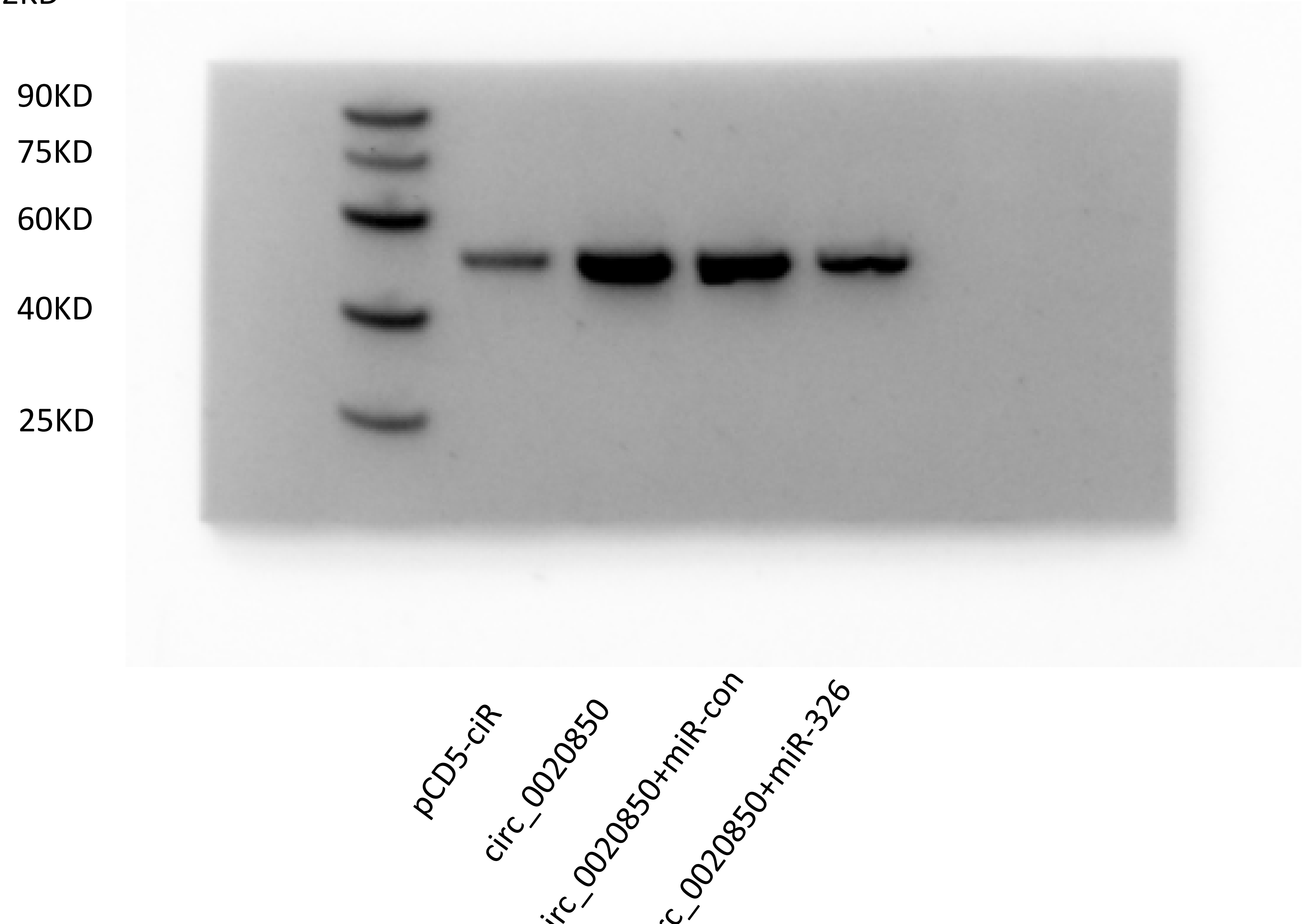

7D-β-actin
42KD

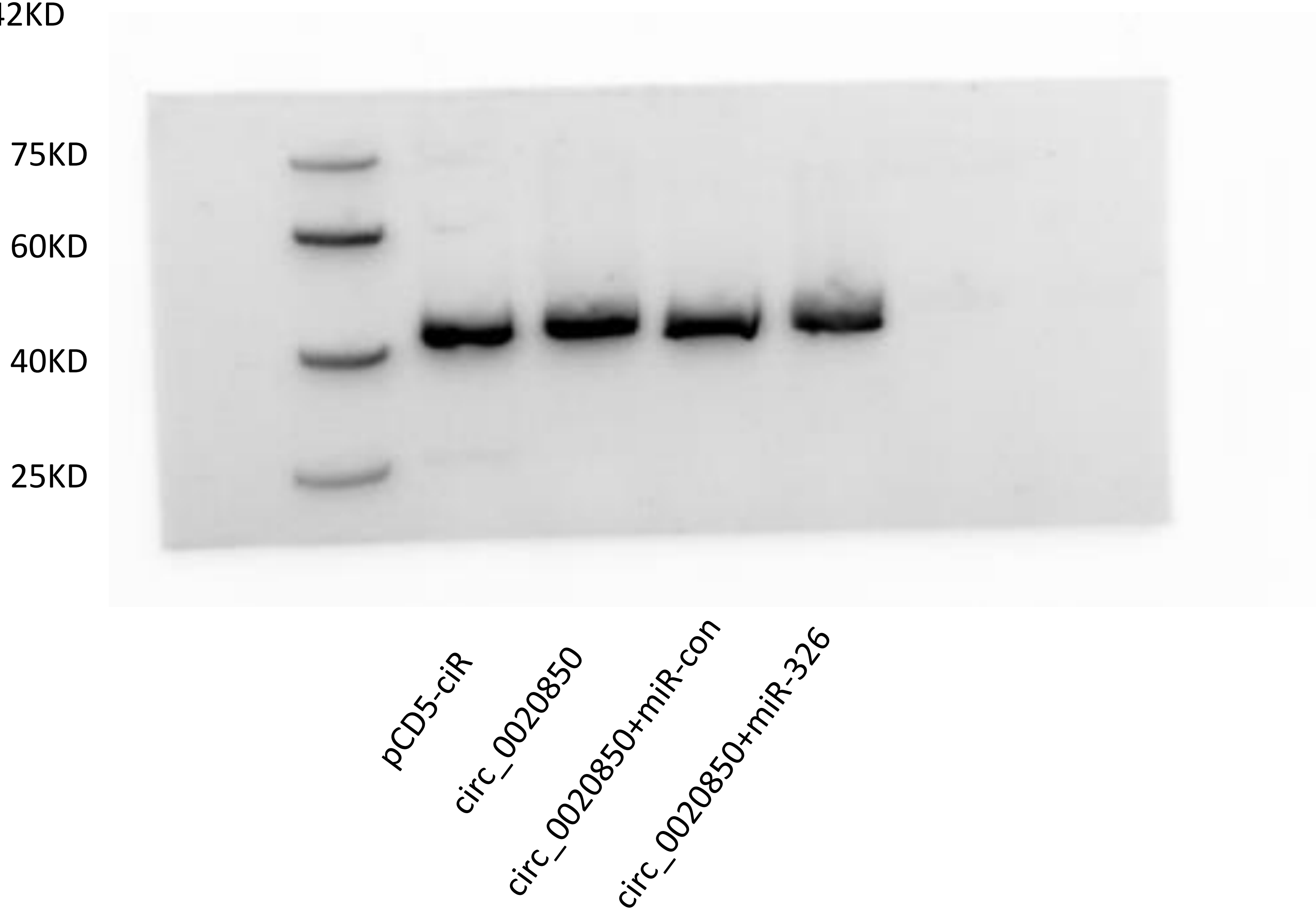

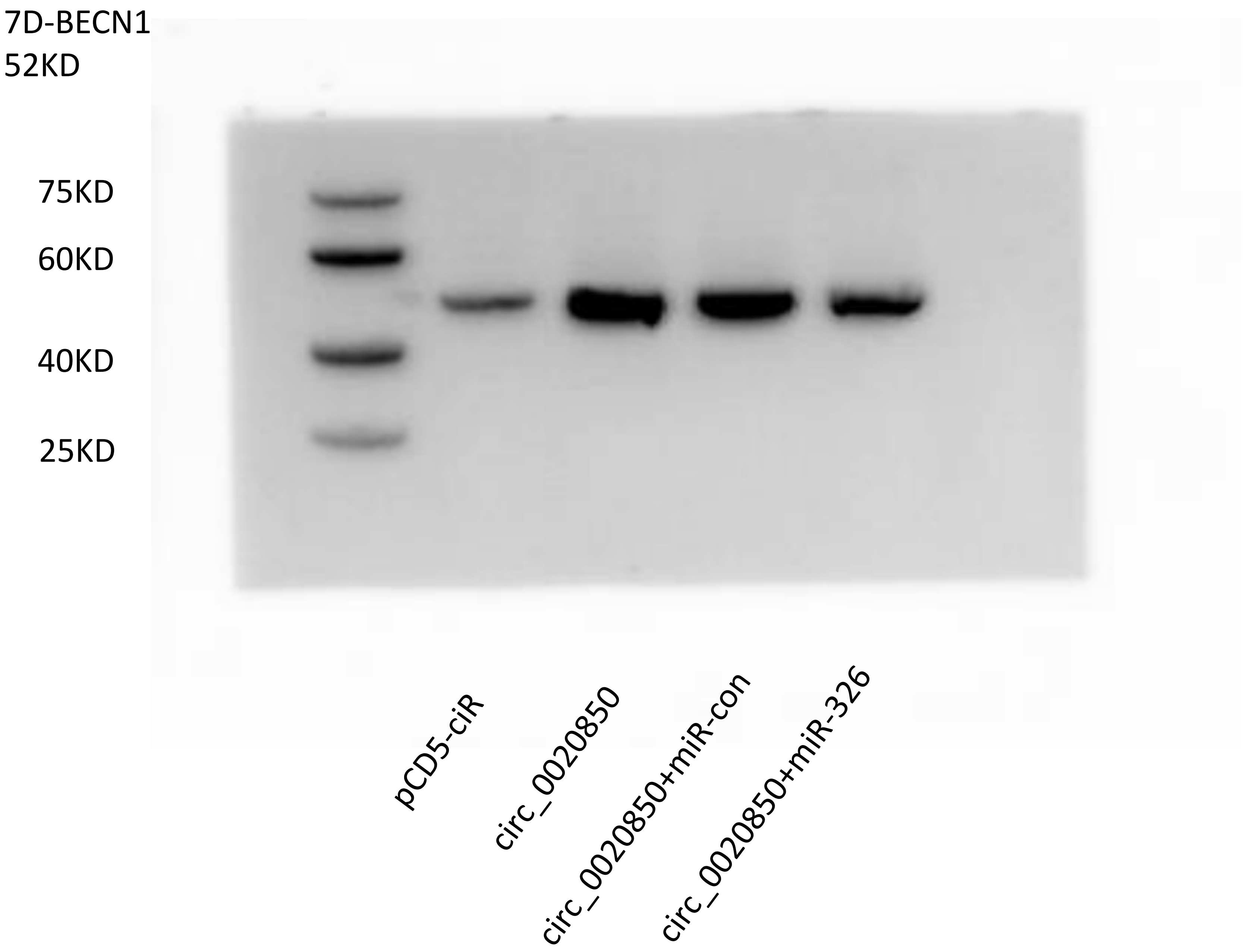
7D-BECN1
52KD
75KD
60KD
40KD
25KD
pCD5-ciR
circ_0020850
circ_0020850+miR-con
circ_0020850+miR-326

8F-β-actin
42KD

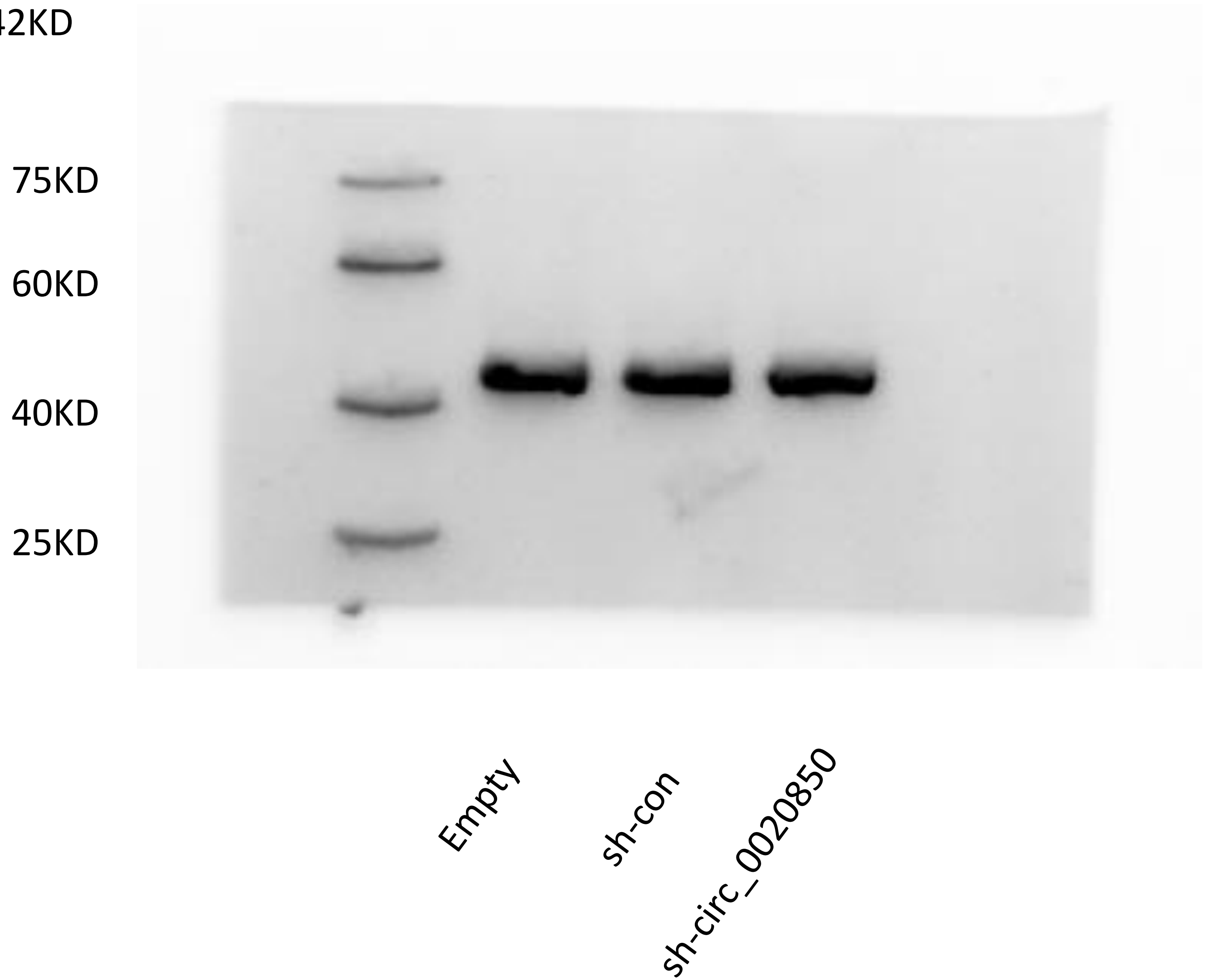

8F-BECN1
52KD

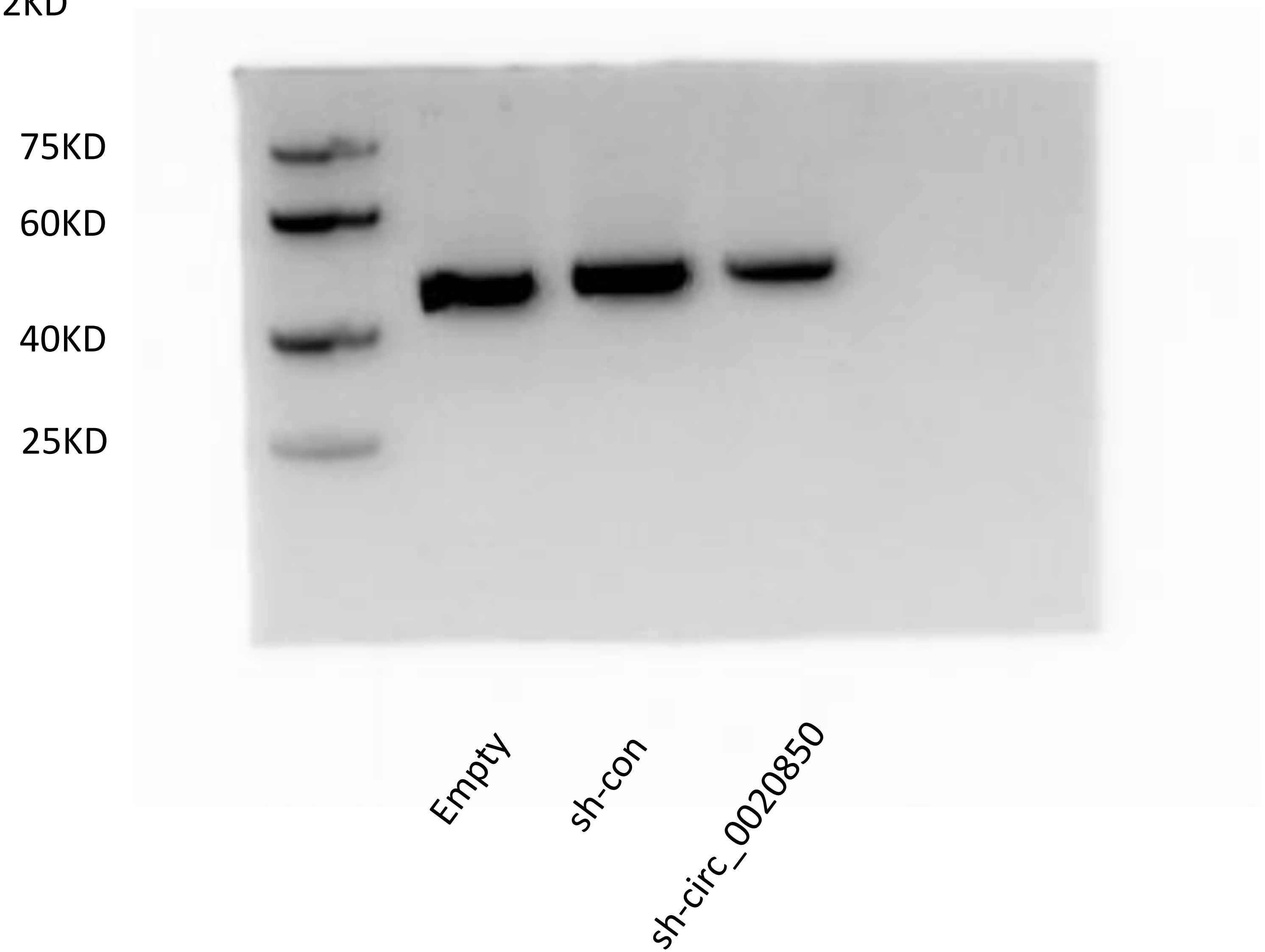

Supplement: Supplementary file 4 — Additional file 4. [file 12957_2021_2480_MOESM4_ESM.pdf]
